# IgG Immune Complexes Inhibit Naïve T Cell Proliferation and Suppress Effector Function in Cytotoxic T Cells

**DOI:** 10.3389/fimmu.2021.713704

**Published:** 2021-08-10

**Authors:** Wissam Charab, Matthew G. Rosenberger, Haridha Shivram, Justin M. Mirazee, Moses Donkor, Soumya R. Shekhar, Donjeta Gjuka, Kimberly H. Khoo, Jin Eyun Kim, Vishwanath R. Iyer, George Georgiou

**Affiliations:** ^1^Department of Chemical Engineering, University of Texas at Austin, Austin, TX, United States; ^2^Department of Biomedical Engineering, University of Texas at Austin, Austin, TX, United States; ^3^Department of Molecular Biosciences, University of Texas at Austin, Austin, TX, United States

**Keywords:** T cell activation proliferation and inhibition, Naive and memory T cells, T cell Fc Gamma Receptors (FcgR), T cell antibody receptors, IgG Immune Complexes (ICs), T cell non-canonical Fc Receptors, Antigen and Antibody Immune Complexes, T cell Fc Receptors

## Abstract

Elevated levels of circulating immune complexes are associated with autoimmunity and with worse prognoses in cancer. Here, we examined the effects of well-defined, soluble immune complexes (ICs) on human peripheral T cells. We demonstrate that IgG-ICs inhibit the proliferation and differentiation of a subset of naïve T cells but stimulate the division of another naïve-like T cell subset. Phenotypic analysis by multi-parameter flow cytometry and RNA-Seq were used to characterize the inhibited and stimulated T cells revealing that the inhibited subset presented immature features resembling those of recent thymic emigrants and non-activated naïve T cells, whereas the stimulated subset exhibited transcriptional features indicative of a more differentiated, early memory progenitor with a naïve-like phenotype. Furthermore, we show that while IgG1-ICs do not profoundly inhibit the proliferation of memory T cells, IgG1-ICs suppress the production of granzyme-β and perforin in cytotoxic memory T cells. Our findings reveal how ICs can link humoral immunity and T cell function.

## Introduction

IgG is the most abundant antibody isotype in serum, and presently all full-length therapeutic antibodies contain an IgG subtype Fc domain (1). When antibodies bind to multivalent antigens, they form immune complexes (ICs) decorated with multiple antibody molecules that can bind with high avidity to Fc gamma receptors (FcγRs) expressed on a wide variety of immune cells, triggering a plethora of well-characterized phenotypes essential for immune homeostasis. IgG ICs are naturally found in sera of healthy subjects, but their presence is often more pronounced in disease states including autoimmunity and cancer (2–7). IC-FcγR interactions can elicit inhibitory or stimulatory signals, contributing to the overall outcome of an immune response (8–10). While FcγRs exclusively bind IgG Fc, non-canonical Fc receptors (nc-FcRs) including certain C-type lectins and mannose receptors, can also bind IgG Fc; however unlike canonical FcRs, nc-FcRs are promiscuous and can interact with IgG, IgA, IgE, and/or IgM Fc (11, 12). Humans express six FcγRs: FcγRI, FcγRIIa/b/c, and FcγRIIIa/b. Engagement of FcγRI, FcγRIIa/c, or FcγRIIIa results in pro-inflammatory responses, whereas FcγRIIb is the sole inhibitory FcγR which plays a role in multiple processes including inhibition of BCR signaling, suppression of inflammation, and the internalization/removal of small ICs by sinusoidal liver endothelial cells (5, 13). While FcγRs are ubiquitous on myeloid cells, their expression on lymphocytes is more restricted. Natural killer and B cells express FcγRIIIa and FcγRIIb, respectively, while about 20% of the human population also express FcγRIIc on NK cells and potentially B cells (8, 9).

Moreover, the expression of antibody receptors (canonical FcRs or nc-FcRs) on T cells is not widely recognized (5, 8–10, 14–19), and it is unknown whether encounter with ICs can directly impact T cell phenotypes. Starbeck-Miller et al. (2014) reported that FcγRIIb is expressed on murine CD8+ memory T cells following bacterial or viral infection and that FcγRIIb engagement suppresses T cell *in vivo* cytotoxicity against peptide-loaded or virus-infected targets (20). In different disease settings including cancer, certain subsets of CD4+ T cells have been reported to express FcγRIII, FcγRII, and/or FcγRI; the ligation of these receptors were reported to enhance interferon-gamma production and cytotoxicity in a subset of human CD4+ T-cells (21, 22). Other studies demonstrated that subsets of CD4+ and CD8+ T cells from HIV-infected patients express FcγRIIa and FcγRIIIa, respectively (23, 24). Very recently, Morris et al. reported that FcγRIIb expression in a subset of effector-memory CD8+ T cells correlates with kidney transplant tolerance following withdrawal from immunosuppression (25). Interestingly, however, experiments in a skin-graft-transplant model indicated that the role of FcγRIIb is independent of IgG antibodies (25).

Here, we examined the effects of well-defined, soluble immune complexes on the phenotypes of human peripheral T cell subsets. We demonstrate that IgG-ICs inhibit the proliferation and differentiation of one subset of naïve T cells but stimulate the division of another naïve-like subset. We utilize RNA-Seq and flow cytometry to further characterize the inhibited and stimulated T cell subsets. The inhibited subset presented immature features similar to those of recent thymic emigrants and non-activated naïve T cells, whereas the stimulated subset exhibited transcriptional features indicative of a more differentiated, early memory progenitor with a naïve-like phenotype. Furthermore, we demonstrate that while IgG1-ICs do not profoundly inhibit the proliferation of memory T cells prevalent in peripheral blood, IgG1-ICs suppress the production of granzyme-β and perforin in cytotoxic memory T cells.

## Methods

All *in vitro* assays with cells from human donors were performed under the supervision of the UT Austin Institutional Review Board (IRB). All animal experiments were performed under the supervision of the UT Austin Institutional Animal Care and Use Committee (IACUC).

### Cells and Culture Reagents

Immune cells were cultured in complete medium. Complete medium comprised RPMI-1640, 10% heat-inactivated FBS (Gibco), 100U/mL penicillin-streptomycin, 0.1 mM non-essential amino acids, 1 mM sodium pyruvate, 1mM HEPES, 1.5mM L-glutamine, and 5.5 μM β-mercaptoethanol. Cancer-patient-derived primary PBMCs were obtained from D. Lee (MD Anderson Cancer Center). Recombinant human IL-2, human IL-4, and murine IL-2 were purchased from STEMCELL Technologies or Biolegend. T cells were cultured in complete medium and typically 10-50 ng/mL rh-IL2. B16F10OVA cells were a kind gift from the Irvine Lab (MIT, MA, USA).

### Flow Cytometry Staining Antibodies and Viability Dyes

Antibodies used for FACS staining (anti-human CD2, CD3, CD4, CD8, CD14, CD19, CD20, CD25, CD28, CD38, CD56, CD57, CD69, CD95, CD127, CD223 (LAG-3), CD366 (TIM-3), TIGIT, CX3CR1, KLRG1, CD279 (PD-1), CD272 (BTLA), TCRαβ, TCRγδ, CD45RA, CD45RO, CD197 (CCR7), Perforin, Granzyme-β, CD62L, CD44, CCR6, CXCR3, and Ki-67) and (anti-murine CD3ε, CD4, CD8α, CD14, CD11b, CD19, CD20, NK1.1, TCRβ, CD62L, CD44, B220, and TER-119) including all Brilliant Violet™ mAbs were purchased from Biolegend. Super Bright™ anti- human CD45RA and CCR7 antibodies were purchased from Thermo. These antibodies were used for purity assessments after magnetic isolation, sorting *via* FACS, or other phenotyping purposes as indicated. For determining cell viability and/or identifying apoptotic cells, Annexin V and SYTOX Green^®^ (Thermo Fisher Scientific) were used. Alternatively, Annexin V and SYTOX Green were employed to correctly set FSC/SSC gates that can similarly distinguish viable *vs.* dead cells instead.

### Recombinant Antibody Expression

Light chain and heavy chain plasmids were constructed for each antibody. The variable domain of heavy and light chain sequences was purchased as gBlocks (Integrated DNA Technologies) and cloned into pcDNA3.4 (Thermo Fisher Scientific) with the appropriate constant domain sequence (WT hIgG, Fc5 hIgG1, hIgE, or, hIgA1). Upon cloning in *E. coli*, the two vectors were transfected at a 3:1 V_L_ : V_H_ ratio into Expi293F^®^ cells using the Expi293 Expression System Kit (Thermo Fisher Scientific) as per the manufacturer’s protocol. 5-7 days later, antibodies were purified using Protein A/G agarose resin (Thermo), buffer exchanged into PBS, and resuspended at 1 mg/mL in PBS. IgA1 and IgE antibodies were purified using peptide M agarose (Invivogen) and Protein L agarose (Thermo), respectively. Human CD32b antibody clone 2B6 is originally a mouse IgG1 antibody capable of distinguishing stimulatory FcγRIIa and inhibitory FcγRIIb (26). To minimize non-specific Fc-staining, a chimera antibody comprising mouse 2B6 variable domains and human constant, aglycosylated (N297D) IgG with human kappa light domains was cloned and expressed. For staining, the 2B6 antibody was conjugated to PE (or FITC) using a dye conjugation kit (ab102918, Abcam). V_L_ and V_H_ sequences for the utilized TNP antibodies (clone 7B4), TYRP1 (Clone 20D7S) antibodies, and anti-human CD32B (Clone 2B6) are provided (**Table S10**).

### Immune Complex Formation

TNP-BSA(25-35) was purchased from Santa Cruz Biotechnology. 10 mg TNP-BSA was resuspended at 2.5 mg/mL in DPBS and buffer exchanged (10-30 kD cutoff) with DPBS to remove preservatives or impurities. To prepare 100 μg of immune complexes (based on antibody amount), 100 μL of 1mg/mL anti-TNP antibodies (anti-TYRP1 or DPBS for isotype and antigen-only controls, respectively) and 40 μL of 2.5mg/mL TNP-BSA (or WT-BSA for some isotype controls) were needed. Importantly, TNP-BSA was added to the antibody solution in 4-5 instalments every 20-30mns with mixing each time and incubating on ice. This approach was taken to encourage the saturation of TNP-BSA molecules with antibodies without having to add excessive molar amounts of mAbs. The resulting mixture was used as a stock of 710 μg/mL ICs (or control treatment).

### PBMC Isolation From Human Blood

About 50-70mL of fresh human blood was drawn from healthy human subjects at the UT Austin University Health Services. Alternatively, 40-60 mL processed blood (source leukocytes) was ordered from a blood bank (Gulf Coast Regional Blood Center, TX) one day before planned experiments. Blood was diluted 1:1 with DPBS, layered over Histopaque-1077 (Lonza), and centrifuged at 800g for 15 mins (no brakes and 3/10 of max acceleration). The PBMC layer was collected by slow pipetting- ensuring no mixing. PBMCs were then washed in cold isolation buffer (2% heat-inactivated FBS, 1 mM EDTA, DPBS) or cold complete medium. Washed PBMCs were then used as the starting material to magnetically isolate various T cell subsets.

### Magnetic Purification of Various Immune Cell Subsets

All T cells were isolated “un-touched” using negative selection kits by STEMCELL. Human Naïve CD8 (19258), Naïve CD4 (19555), Memory CD4 (19157), Memory CD8 (19159), Total CD4 (17952), Total CD8 (17953), and Total CD3+ T (17951) cell kits were routinely used. When using other brands, caution must be taken before use to ensure the absence of FcR-blocking reagents. Negative-selection kits enrich for all αβ T cell subsets by depleting markers associated with “unwanted” cells (e.g., CD14, CD16, CD19, CD20, CD34, CD36, CD56, CD61, CD66b, CD123, TCRγ/δ, glycophorin A). Naïve T cell negative-selection and Memory T cell negative-selection kits deplete markers to enrich for αβ T cells in addition to depleting other markers associated with either memory/activated (CD45RO, CD57, CD94, CD244, CD25) or naïve T cells (CD45RA), respectively. Mouse naïve T cells (19848), Total B cells (19854), and Total T cells (19851) kits were also used. CD14+ monocytes and CD56+ NK Cells were isolated from peripheral blood using positive selection kits (17858 and 17855, respectively). All labeled cells were separated using the EasySep™ or Big Easy™ magnet (STEMCELL).

### CellTrace® Violet Staining

Isolated T cells were washed in DPBS 3X (to remove soluble proteins) and resuspended in DPBS at 1M/mL cells. 1 uL of freshly prepared CellTrace Violet solution (5 mM in DMSO) was added for every 1mL of T cell suspension. Resuspended cells were placed in ultra-low-attachment flasks (Corning #3814) to minimize adhesion and cell loss. Flasks were then incubated for 20-30 minutes in a 37°C 5% CO_2_ incubator. Afterwards, complete medium was added to wash cells (4X-5X the original staining volume) and the flask was incubated in the dark at RT for 5 minutes. Cells were then pelleted and re-suspended in pre-warmed complete medium.

### Preparing T Cells for *In Vitro* Functional Assays: Activation, IC-Incubation, and Culture

Upon isolating various T cell subsets, cells were labelled with CellTrace^®^ Violet as described above. Labelled cells were suspended in pre-warmed complete medium (10-20 ng/mL rh-IL2) at 0.75-1.5M/mL (~1M/mL most commonly). Cells were then pre-incubated with immune complexes or control treatment at 37°C, 5% CO_2_ for 2 hours (do not wash). The concentration of ICs or control treatment used was typically 30-50μg/mL unless otherwise specified. Subsequently, IC-treated T cells were activated using *washed* anti-human CD3/anti-CD28 Dynabeads^®^ (30uL/1M T cells, Thermo). Empty plate wells were routinely filled with DPBS to minimize evaporation. Three to four days later (or the day of bead removal), cultured wells were replenished with pre-warmed complete medium (10-20 ng/mL rh-IL2) containing the IC/control treatment (optional). Dynabeads were removed magnetically at t= 60 ± 12 hrs. post-activation. Instructions for washing and removing Dynabeads are provided below. On days 5-7 post-culture, (a) culture supernatant was collected for cytokine release assays and/or (b) cultured cells were washed, stained for viability or other markers of interest, and scanned by FACS.

#### Washing Dynabeads Prior to Use

A predetermined volume of Dynabeads stock solution (e.g. 0.5 mL) is resuspended in complete medium (e.g. 1.5mL for a total of 2mL volume) in 2mL Eppendorf tubes. The beads are gently mixed using a P1000 pipet. The 2mL tube is then placed in a magnet (DynaMag™-2) for 1-2 minutes. While in the magnet, without moving the tube, a P1000 pipet is used to remove the supernatant completely. The tube is then removed from the magnet and the beads are washed with a fresh 2mL aliquot of complete medium. This process is repeated thrice (four total washes). Finally, beads are resuspended in their same original volume (e.g. 0.5mL) of culture media. For washing, complete medium (containing 10% FBS) is recommended over DPBS since the latter can lead to bead loss due to non-specific bead sticking to plastic surfaces (e.g. tube or pipet tips) when in DPBS alone.

#### Removing Dynabeads After Activation

Anti-human CD3/anti-CD28 Dynabeads were removed magnetically at t= 60 ± 12 hrs. post-activation. Briefly, cells were transferred to 2mL Eppendorf tubes and gently resuspended using a P1000 pipet directly before inserting the tubes into a magnet (DynaMag™-2) for 1-1.5 minutes. Mixing prior to this step and not exceeding 1.5 minutes is important to minimize cell loss upon bead removal (i.e. T cells can stick to the beads). While in the magnet, without moving the tube, a P1000 pipet is used to remove the supernatant (containing the T cells) completely. Beads are left behind in the tube. Beads can be washed again with a small volume of warm T cell media (e.g. 100-200uL) to recover sticky T cells and combine them with first cell fraction. The same procedure must be followed for all samples. Dynabead-removed, cells fractions are placed in fresh plates and returned to the incubator.

## *In Vitro* Cytokine Release Experiments

Cytokine release was assayed using a bead-based multiplex assay panel, using fluorescence–encoded beads analyzed by a flow cytometer (BD LSR Fortessa) as instructed by the assay manufacturer (LegendPlex; Biolegend Catalog# 740722). Naïve CD8+ T cells from five unique human donors (017, 050, 052, 054, and 058) were labeled with CellTrace Violet (if applicable for proliferation experiments); activated with anti-CD3/anti-CD28 Dynabeads; and cultured with IgG1-ICs or controls (Fc5 IgG1 ICs, monomeric IgG1, and DPBS in duplicate) as described above (see *Preparing T Cells for In Vitro Functional Assays: Activation, Immune Complex Incubation, and Culture*). Non-activated T cells from two donors (052 and 058) were also cultured as non-stimulated controls. Briefly, after 5.5 days in culture, cells were pelleted to collect the culture supernatant (first in a gentle spin at 250g to remove cells; then supernatant was centrifuged at 2000g to remove any debris). Note that several dilutions of a sample may be required to ensure that a reading falls within the linear dynamic range of the assay. In our experiments, a dilution factor of 3 was appropriate for all naïve CD8 samples.

## t-SNE Analysis of Activated Naïve T Cells

Naïve T cells from three unique human donors (052, 057, and 058) were labeled with CellTrace Violet; activated with anti-CD3/anti-CD28 Dynabeads; and cultured with IgG1-ICs (in triplicate) or controls (Fc5 IgG1 ICs, monomeric IgG1, and DPBS in duplicate) as described above (*Preparing T Cells for In Vitro Functional Assays: Activation, Immune Complex Incubation, and Culture*). Non-activated T cells were also cultured as non-stimulated controls. Briefly, after 5.5 days in culture, cells were washed then stained on ice using two panels of antibodies (referred to herein as Panel A and Panel B; **Table S3**). FlowJo’s tSNE plug-in was used to analyze the data (N=33 wells/panel). Prior to tSNE analysis, viable single cells were gated (FSC- and SSC-A/H gates). FlowJo’s “DownSample” plug-in was used to sample an equal number of viable single cells across all samples (e.g. 7790 events). The down-sampled populations were then concatenated into a single file. Next, the tSNE plug-in was utilized to analyze the concatenated file by assessing only the following parameters for each panel: Panel A: CD57, KLRG1/CX3CR1, LAG-3/TIM-3/TIGIT, CCR7/CD62L/CD28, CD25/CD38/CD69, PD1, and BTLA. Panel B: CD127, CD45RA, CD45RO, CD95, CCR7/CD62L, and CD28. CellTrace was not included at this stage to prevent the algorithm from binning cells based on proliferation status; instead, CellTrace staining was assessed *after* tSNE analysis on the resulting tSNE “islands” to distinguish undivided naïve T cells (Generation1; Gen1) and proliferating progeny. Both panels were processed the same way using default settings (Perplexity=30; Iteration= 1000). The resulting tSNE file was further analyzed on FlowJo and gated populations/island memberships (frequency and MFI) were plotted on GraphPad.

## Analysis of *In Vitro* T Cell Proliferation Experiments

CellTrace comprises a cell-permeant, non-fluorescent ester of an amine-reactive fluorescent molecule which diffuses into cells. Upon cell entry, cellular esterases convert the molecules to a fluorescent form that covalently binds proteins. This long-term retention allows reliable tracking of each cell division of every generation of daughter cells which “inherit” ~50% of fluorescently labelled proteins from the parent cell. Using FACS, each cell generation could be identified by its relative fluorescent intensity allowing the calculation of variables that describe proliferation (27) including: (a) the percentage of input cells that have not divided (i.e. “% undivided” and referred to herein as “Φ_1_” for brevity); and (b) the percent of each T cell generation that has undergone any number of divisions (represented by θ_i_, where “i” is the generation number). Plotting “θ_i_ *vs.* i” essentially yields a mathematical representation of the FACS-derived proliferation curves. θ_i_ (or 100-Φ_i_) is % division (or 100-% undivided) for a particular T cell generation (i). For instance, θ_1_ is equal to (100- Φ_1_) where Φ_1_ is % undivided. Φ_1_ can be calculated using the following equation:

$$\Phi_{1}=\frac{{\text{X}}_{1}}{{\text{X}}_{1}+\Sigma_{i=1}^{D}\frac{{\text{X}}_{i}+1}{2}}$$

where D is the total number of observed divisions (number of peaks -1) and X_1_ is the frequency of the 1^st^ peak gated in FlowJo. To calculate Φ_2_, the same equation is used but “X_1_” would need to be recalculated by excluding generation 1 before gating. To calculate Φ_3_, the same equation is used but “X_1_” would need to be recalculated by excluding generations 1 and 2 before gating- so on and so forth. A template excel sheet that automatically calculates and plots θ_i_ (or Φ_i_) *vs.* i is provided as a supplemental attachment. Alternatively, % undivided (Φ_1_) and other proliferation parameters can be calculated by the proliferation plugin in newer versions of FlowJo.

### Effector Memory T Cell Enrichment

Isolated human PBMCs were followed by Total T cell isolation as described above. Total human T cells were then stained for CCR7 and impurities (CD14/CD19/CD56/TCRγδ). Stained cells were then sorted by FACS for CCR7(-) effector T cells (effector memory and terminal effector memory) for cytotoxicity assays.

### Intracellular Production of Granzyme and Perforin

Sorted effector T cells were cultured in complete medium (30 ng/mL rhIL2) at 1-2M/mL in tissue culture plates. Before the next step, sorted cells can be allowed to recover (optional) overnight at 37°C, 5% CO_2_. Cells were then pre-incubated with immune complexes or control treatment at 37°C, 5% CO_2_ for 2 hours (do not wash). Subsequently, IC-treated T cells were activated using *washed* anti-human CD3/CD28 Dynabeads^®^ (30uL/1M T cells, Thermo). Empty plate wells were routinely filled with DPBS to minimize evaporation. 48 hours later, T cells were washed with DPBS, fixed, and permeabilized with a kit (catalog # 00-5523-00 eBioscience). Fixed and permeabilized cells were stained for Ki-67, Perforin, and Granzyme-β. The concentration of ICs or control treatment used was typically 30-50μg/mL unless indicated otherwise.

### RNA-Seq Analysis of Inhibited and Stimulated Naïve CD8+ T Cells

Proliferation experiments were set up for three unique human donors (017, 050, and 054) as previously described (*Preparing T Cells for In Vitro Functional Assays: Activation, IC-Incubation, and Culture*). Briefly, CellTrace-labelled naïve CD8+ T cells were activated and incubated with 50μg/mL IgG1-ICs (017, 050, and 054; n=3) or Fc5 IgG1-ICs (017 and 054; n=2) in technical duplicates. Dynabeads were removed at t= 60 hrs. After 5.5 days in culture, IgG1-IC-treated T cells were sorted based on CellTrace fluorescence into two fractions: Inhibited T cells (InhT) and Stimulated T cells (StimT) as summarized in **Figure 4E**. The sorted populations ranged in number (1.7x10^5^ – 1.9x10^6^ cells/replicate/donor). Fc5-IgG1-IC-treated T cells (Fc5T) were not sorted and served as controls. Total RNA was extracted from InhT, StimT, and Fc5T samples (Qiagen). RNA samples were then forward processed by the Genomic Sequencing and Analysis Facility (GSAF) at UT Austin. Briefly, mRNA was isolated (poly-A capture) and stranded libraries were created (dUTP Method; NEB NGS kit) for paired-end sequencing (PE75, NextSeq 500). If any replicate for a particular donor failed to create a quality library, GSAF equally amplified all samples from that donor (6 PCR cycles) and re-attempted library creation. The total number of read pairs ranged from 2.7x10^7^ to 6.5x10^7^/sample. Subsequently, adapters were trimmed from the reads using Cutadapt (28) and then aligned using HISAT (29) to the human genome version hg38. Read counts were generated by FeatureCounts (30) using parameters for fractional counting. Read counts were normalized by library size using DESeq2 (31). Quality and concordance of sample replicates were evaluated using MultiQC (32) and by performing the principal component analysis (PCA). A majority of the samples showed greater than 90% mapping rate. Of all 16 samples, two (InhT/Donor 017/Replicate B and InhT/Donor050/Replicate A) were outliers based on PCA and also showed significantly lower mapping rates; therefore, these samples were eliminated from downstream analysis. Differential gene expression was calculated between sample pairs using DESeq2 at FDR corrected p-value < 0.05 to identify differentially expressed genes (DEGs). Gene expression as transcripts per million was calculated using the TPMCalculator tool (33) (https://github.com/ncbi/TPMCalculator) at default settings. To identify overlapping pathways associated with DEGs, the MSigDb database (34) was queried using the online GSEA tool (http://www.gsea-msigdb.org/gsea/msigdb/annotate.jsp). Furthermore, gene set enrichment analysis was performed using the GSEA software (GSEA v4.0.3 for Windows) and GSEA instructions (https://www.gsea-msigdb.org/gsea/doc/GSEAUserGuideFrame.html). Default settings were used (e.g. 1000 gene set permutations and “Signal2Noise” ranking metric). As recommended by GSEA for the standard pipeline (without manual pre-ranking of genes), hits with an FDR q-value <0.25 were considered enriched. In addition to the gene sets available in the GSEA database (e.g. H, Hallmark; or C2, Curated Gene Sets), another dataset was assessed. This dataset was curated from previous reports of genes that were enriched or differentially expressed in dysfunctional T cell states (35, 36). or by FcγIIb+ T cells (25).

### Gene Expression Analysis of Previously Published Datasets

Since exons of FcγRIIA, FcγRIIB, and FcγRIIC are highly similar, segments of their transcripts are virtually indistinguishable from one another. Thus, all FcγRII reads aligning to one of these isoforms were grouped together and counted only once. The same was done for FcγRIII (FcγRIIIA and FcγRIIIB). Annotated scripts used for RNA-seq and microarray data analyses are deposited to GitHub (https://github.com/haridh/Georgiou_Lab_Collab).

#### Bulk RNA-Seq

RNA-seq data were downloaded from GEO (GSE63147) and the EMBL-EBL data repository (https://www.ebi.ac.uk/). Adapters were trimmed from the reads using Cutadapt (28) and then aligned using HISAT (29) to the human genome version hg38. The total number of aligned reads was ~30-60M/sample. Read counts were generated by FeatureCounts (30) using parameters for fractional counting. Read counts were normalized by library size using DESeq2 (31). Gene expression for specific genes was calculated as - DESeq2 normalized read counts/total gene length in kilobases. For e.g., FCGR2 gene expression was calculated as DESeq2 Normalized reads (FCGR2A + FCGR2B + FCGR2C)/Gene Length (FCGR2A + FCGR2B + FCGR2C).

#### RNA Microarray

Microarray data from GSE12589 were analyzed using GEOquery R package (37). Values corresponding to Log10 Normalized signal (Infected/Control) for specific genes were extracted as described in the R package vignette. Values were then plotted on Microsoft Excel.

#### Sc-RNA-Seq

For single cell RNA-Seq, data from GSE98638 were analyzed to calculate the total counts of FCGR reads per million reads (cpm) for each cell within each cluster deduced by the RNA transcriptome analysis done by Zheng et al. (38). Reads for FCGR2A, FCGR2B, and FCGR2C were not distinguished, combined, and represented as FCGR2 reads. The same was done for FCGR3A and FCGR3B.

### IC Binding by Confocal Microscopy

Fluorescent ICs were prepared by conjugating 100 μg TNP-BSA to Cy5 (Abcam # ab188288). Cy5-conjugated TNP-BSA was then buffer exchanged (10-30 kD cutoff) with DPBS to remove free Cy5. Fluorescent ICs were then formed by mixing 50 μg Cy5-TNP-BSA with either WT- or Fc5- anti-TNP IgG1 (160 μg at1 mg/mL). Due to material availability, the final concentration of fluorescent ICs incubated with cell for IC-binding experiments (~5 μg/mL) was lower than that used for functional experiments (30-50 μg/mL). Thus, for brighter images, more fluorescent ICs may be generated and used at higher concentrations.

Total T cells, B cells, and monocytes were negatively selected as previously described. Cells (5-10 million/condition) were pre-blocked (complete medium containing 20% human AB serum, 20% mouse serum, and 0.1% sodium azide) on ice for one hour. Without washing the block solution, fluorescent ICs were added and cells were incubated on ice for an additional two hours. Afterwards, antibodies targeting lineage markers (CD3, TCRαβ, CD14, and/or CD19) and FcγRs (CD16 PE clone 3G8; CD32 PE clone FUN-2; and/or BV421 CD64 clone 10.1) were added to the cells (with blocking solution and ICs) for 40 minutes. Cells were washed thrice and fixed (BD Bioscience). Fixed cells were visualized by a confocal microscope (Zeiss LSM 710/Elyra S.1.). Zen Lite Blue was used for analysis (Black= 3; Gamma= 1; White 64,313). All confocal images were equally enhanced (+40% contrast; +40% brightness) for clarity.

### FcγR Expression by Flow Cytometry

For FcγR staining, anti-human CD32 (clone FUN2), MOPC-11 (μIgG2b isotype), anti-human CD64 (clone 10.1), anti-human CD16 (clone 3G8), MOPC-21 (μIgG1 isotype), Clone MG1-45 (μIgG1 isotype) were purchased from Biolegend. Isotype control brightness and final concentrations were matched to that of FcγR-staining mAbs before staining. Cells were pre-blocked (complete medium containing 20% human AB serum, 20% mouse serum, and 0.1% sodium azide) on ice for one hour before antibody staining. To compare cell staining on the surface only *vs.* surface and intracellularly, cells were washed four times after extracellular FcγR-staining. Then half the cells were followed by fixation/permeabilization (BD Bioscience Catalog# 554714), and intracellular staining with either FcγR-staining antibodies or DPBS only.

For competitive binding experiments, to ensure that observed fluorescence shifts after anti-CD32b staining were indeed due to FcγRIIb binding, aliquots of anti-CD32b were pre-incubated with either soluble GST-tagged-human FcγRIIb or DPBS. The soluble receptors (2-3 mg/mL) were added in molar excess (~100 μg GST- FcγR in ~20uL DPBS) to the staining antibody (~2 μg in ~10uL) and incubated on ice for at least 30 minutes. Using those stocks, equal molar amounts of antibody were used to stain cells.

## Data Analysis and Statistics

Data were processed using Microsoft Excel, GraphPad Prism, FlowJo V10, Zeiss Zen Lite, and Amnis IDEAS Software. Figures were created with InkScape and some clip arts were created using Adobe Illustrator. For two-condition comparisons (e.g. IgG1-ICs *vs.* negative controls) across individual donors, p-values were calculated using unpaired, two-tailed t-tests adjusting for multiple comparisons using the Sidak-Holm correction. Consistent standard deviation was not assumed unless an experiment pertained to a single-well assay. For two-condition, aggregate comparisons across all donors, p-values were calculated using the Mann-Whitney U statistical test. For multiple-condition, aggregate comparisons across all donors, p-values were calculated using the ordinary one-way ANOVA statistical test. GraphPad Prism was used for these statistical tests. Error bars in figures represent the standard error of the mean (SEM). For statistical analyses of RNA-Seq data, nominal p-values, adjusted p-values, and FDR-adjusted q-values were reported as calculated by DESeq2 or, where applicable, GSEA. For p-values of the HCMV microarray, the paired, nonparametric Wilcoxon-signed rank test was used by the authors of that dataset (39).

## Results

### IgG1 Immune Complexes Inhibit Naïve T Cell Proliferation but Stimulate a Subset of Their Progeny

**Figure 1:**
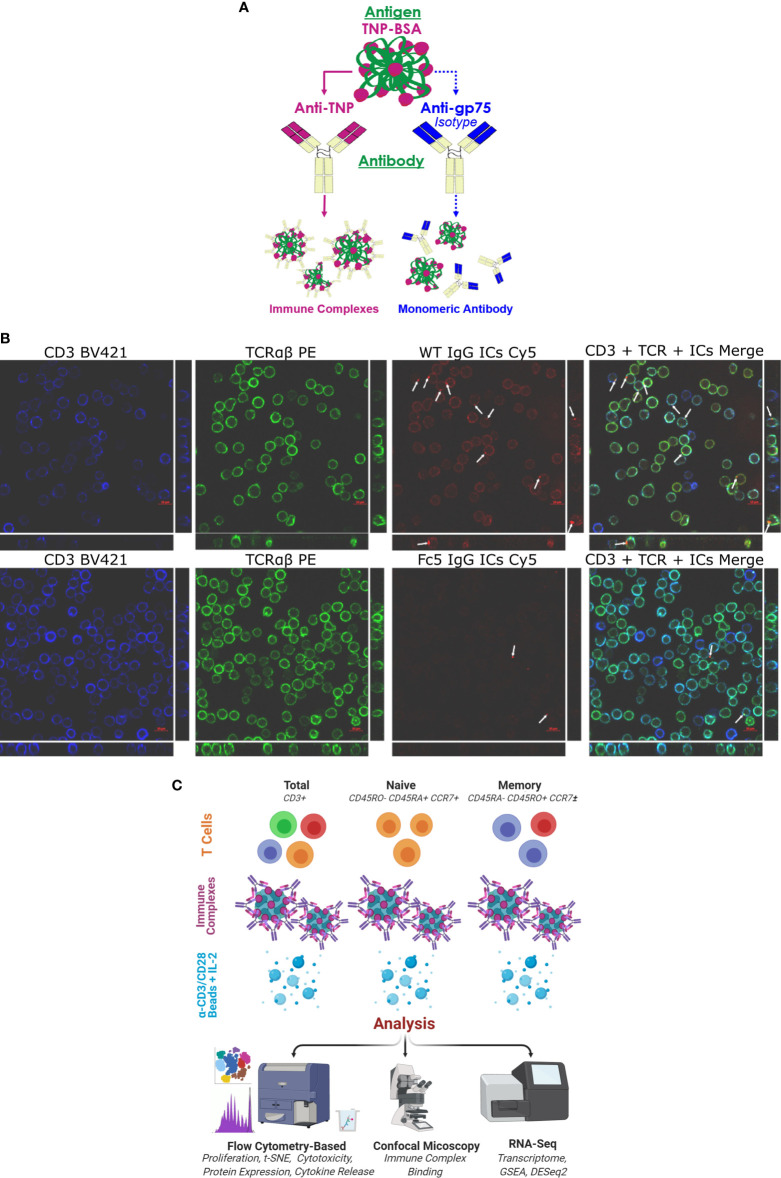
IgG1 Immune Complexes Bind T cells **(A)** Overview of antibody/antigen preparations added to activated T cells. **(B)** Confocal microscopy of purified T cells stained with anti-CD3 (blue), anti-TCRαβ (green), and fluorescent ICs (red). T cells were incubated with either WT IgG1-ICs or Fc5 IgG1-ICs. White arrows show fluorescent IC clusters. Large and strip images pertain to the x-y and y-z/x-z planes, respectively **(C)** Schematic overview of experimental protocol. Various T cell subsets are incubated with antibody/antigen preparations; activated with anti-CD3/anti-CD28 beads; and cultured with recombinant interleukin-2 (IL-2). IC-binding experiments were performed on ice using negatively selected, resting CD3+ T cells.

Well-defined immune complexes (ICs) were formed using trinitrophenol-conjugated bovine serum albumin (TNP-BSA; 25-35 TNP-moieties/BSA molecule) as the antigen and monoclonal anti-trinitrophenol antibodies to form TNP-BSA-anti-TNP ICs (40, 41). We examined the effects of ICs formed using antibodies of different isotypes (IgA1, IgE, and IgG1; IgA1-, IgE- and IgG1-ICs respectively). Additionally, we generated ICs comprising an engineered aglycosylated IgG1 Fc (Fc5 IgG1) that selectively binds FcγRI with high affinity but presents no binding to all other FcγRs (42). Negative controls comprising monomeric antibody (e.g. TNP-BSA mixed with anti-human TYRP-1 antibodies, isotype control) were also utilized (**Figure 1A** and **Table S1**). As expected, wild-type (WT) IgG1-ICs bound to purified low-affinity FcγRs with EC50s that were >10-100-fold lower relative to monomeric IgG1. Monomeric Fc5 IgG1 and Fc5 IgG1-ICs bound only to FcγRI and not to any of the low affinity receptors. Likewise, IgA1-ICs and IgE-ICs, but not IgG1-ICs, bound only FcαRI (IgA FcR) and FcϵRI (IgE FcR), respectively (**Figure S1**).

**Figure 2:**
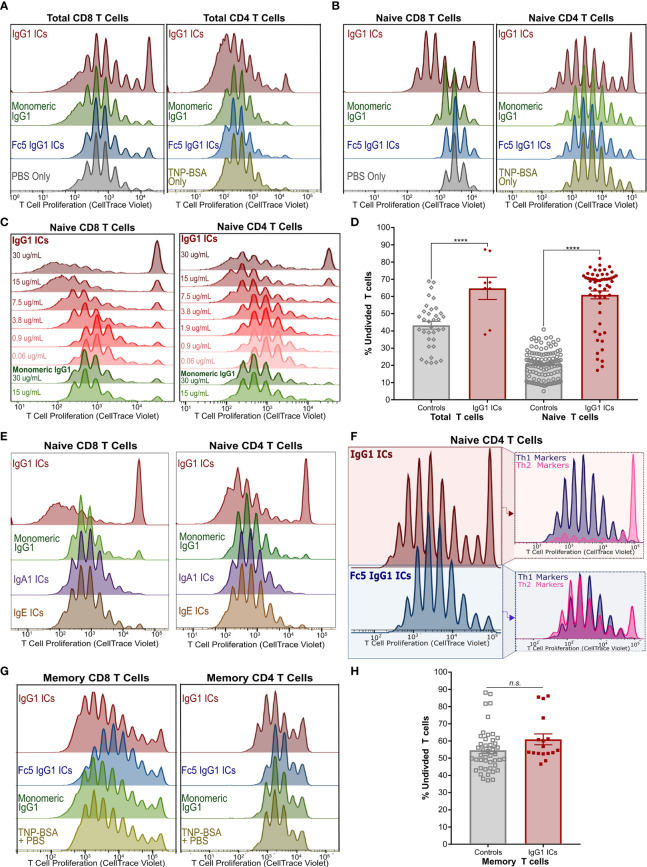
IgG Immune Complexes Inhibit Naïve T Cell Proliferation but Stimulate a Subset of Their Progeny **(A)** Proliferation of activated CellTrace-stained Total CD8+ (left) and CD4+ (right) T cells incubated with IgG1-ICs or indicated controls (representative of 3 independent experiments/donors). **(B)** Proliferation experiment described in **(A)** with purified naïve CD8+ (left) and naïve CD4+ (right) T cells (representative of 8 independent experiments using 7 unique human donors for CD8+; 5 independent experiments/donors for CD4+). **(C)** Proliferation of activated naïve CD8+ and CD4+ T cells incubated with varying concentrations of IgG1-ICs (various shades of red) or monomeric IgG1 (green). **(D)** Summary of total and naïve T cell proliferation data **(E)** Proliferation experiments described in **(A)** with representative results for IgA1-ICs and IgE-ICs. **(F)** Activated naïve CD4+ T cells incubated with IgG1-ICs or Fc5 IgG1-ICs, cultured for 5-7 days, and stained with anti-CXCR3 and anti-CCR6 mAbs to examine Th1-like (CXCR3+ CCR6-) and Th2-like (CXCR3- CCR6-) phenotypes (representative of 4 independent experiments/donors). **(G)** Proliferation experiment described in **(A)** performed using enriched memory CD8+ T cells (representative of 3 independent experiments/donors for CD8+; 4 independent experiments/donors for CD4+). **(H)** Summary of memory T cell proliferation data. “****” indicates p-values < 0.0001 (one-way ANOVA). “n.s.” stands for statistically not significant. A tabulated summary of all proliferation experiments (N=26) for individual donors is provided in **Table S2**.

Confocal microscopy demonstrated that IgG1-ICs, but not FcγRI-selective Fc5 IgG1-ICs, were localized on resting CD3+ TCRαβ+ T cells (**Figure 1B**). Various T cell subsets (total, naïve, or memory CD4+ and CD8+ T cells) were negatively selected (**Figure S2**; see *Methods* for description of depleted markers) and activated by incubation with anti-CD3/anti-CD28 magnetic beads for 60 hours. The cells were incubated with IgG1-ICs, control (Fc5 IgG1-) ICs, or monomeric IgG1 at t=0 and cultured for a total of 5-7 days (**Figure 1C**). Cell proliferation was evaluated using CellTrace^®^ Violet, which is a cell-permeable fluorescent dye that covalently binds free amines. Compared to negative controls, IgG1-ICs, but not Fc5 IgG1-ICs or monomeric IgG1, suppressed the proliferation of total CD8+ T cells by 61% ± 16% (N=3 donors, p-value <0.01) and CD4+ T cells by 56% ± 24% (N=3 donors, p-value <0.01) (**Figure 2A** and **Table S2**).

We next examined the effects of ICs on the proliferation of activated naïve CD8+ and naïve CD4+ T cells. Incubation with IgG1-ICs, but not with control ICs or monomeric IgG1, induced (1) profound inhibition of both naïve CD8+ and CD4+T cells; and (2) partial stimulation of a subset of their progeny (**Figures 2B** and **S3A**). Inhibition and stimulation were dependent on IC concentration for both naïve CD8+ and CD4**+** T cells (**Figure 2C**). The quantification of proliferation for total and naïve T cells is summarized in **Figure 2D** and **Table S2**. While the magnitude of the observed effects were donor-dependent, IgG1-IC inhibition of naïve T cell proliferation was clearly evident in the “percent undivided” naïve CD8+ T cells (mean increase in percent undivided cells: 200% ± 74%; range:110%-370%; 8 independent experiments; 7 unique donors; p-value <0.01) and CD4+ T cells (mean increase in percent undivided cells: 110% ± 54%; range: 50%-190%; 5 independent experiments; 5 unique donors; p-value <0.01) compared to negative controls (**Table S2**). Conversely, as measured by percent division compared to controls, statistically significant stimulation (p-value <0.05) was observed in 42% ± 33% of CD4+ and 57% ± 20% CD8+ T cell generations, respectively (**Figure S3A**). In terms of overall number of divisions, compared to negative controls, this stimulatory effect was more pronounced in CD8+ T cells (median 7.5 *vs.* 9 divisions; p-value <0.05) than CD4+ T cells (median 9 *vs.* 10 divisions; p-value: 0.18). Neither monomeric IgG1 nor immune complexes formed using IgA1, IgE, or FcγRI-selective Fc5 IgG1 antibodies had any effect on proliferation (**Figures 2E** and **S4**), indicating that the observed effects on naïve T cells are mediated by an IgG receptor, that does not bind IgA or IgE, and requires IgG engagement in the context of multivalent immune complexes.

**Figure 3:**
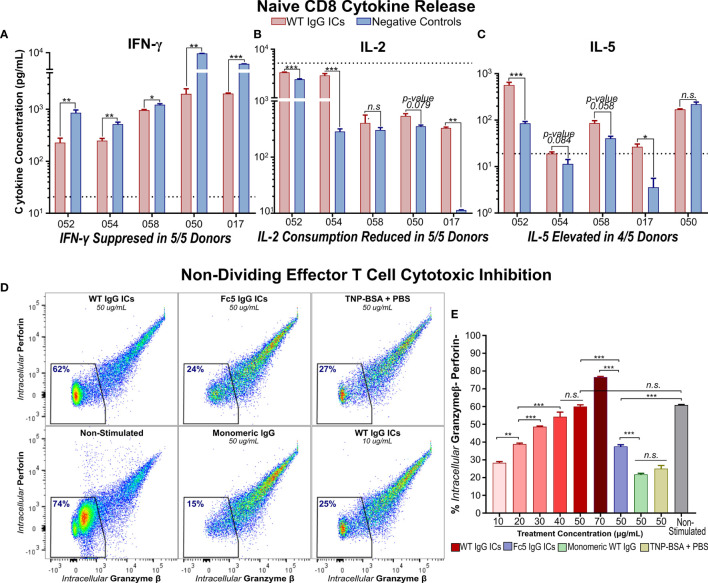
IgG Immune Complexes Suppress IFN-γ Secretion by Activated Naïve CD8+ T cells and Inhibit Effector Molecule Production by Cytotoxic Memory T Cells **(A–C)** IFN- γ, IL-2, and IL-5 secretion by activated naïve CD8+ T cells incubated with (WT) IgG1-ICs or controls (Fc5 IgG1-ICs, monomeric IgG1, or PBS). Dashed line represents the average cytokine concentration in non-activated cultures from two donors **(D)** Flow cytometry analysis of intracellular production of perforin (Prf) and granzyme-β (Gzmβ) in non-proliferating effector memory T cells. Purified CCR7- T cells were either (i) unstimulated and untreated or (ii) activated and incubated with IgG1-ICs or indicated controls. The percent of Gzmβ(-) Prf(-) cells is indicated in blue. **(E)** Gzmβ/Prf production as a function of IgG1-IC concentration or 50μg/mL negative controls. The assay was run in triplicate wells. **(A–E)** Error bars represent the standard error of the mean (SEM). Asterisks ^*^, ^**^, and ^***^ indicate p-values < 0.05, p-values < 0.01, and p-values < 0.0001 respectively. “n.s.” stands for statistically not significant.

Furthermore, phenotypic differences were observed when activated naïve CD4+ T cells were incubated with IgG1-ICs. Namely, IgG1-ICs selectively inhibited CCR6- CXCR3- CD4+ [Th2-like (43)] T cells while having a very mild stimulatory effect on CCR6- CXCR3+ [Th1-like (43)] cells (**Figures 2F** and **S5**). Moreover, IgG1-ICs had no profound effects on memory CD8+ or CD4+ T cell proliferation (3 independent CD8+ experiments, p-value >0.05; 4 independent CD4+ experiments, p-value > 0.16) (**Figures 2G, H** and **Table S2**). For all naïve and memory T cell experiments, cell viability was on average >80% at the end of the experiment indicating that incubation with ICs did not result in cell death (**Figure S6**). In addition to proliferation, we also examined cytokine release of activated naïve CD8+ T cells incubated with ICs. Incubation with IgG1-ICs suppressed interferon-gamma (IFN-γ) secretion and interleukin-2 (IL-2) consumption (as compared to cytokine levels for cultured but non-activated T cells) as may be expected in light of the increased fraction of undivided cells (**Figures 3A–C**; n=5 donors). Incubation with IgG1 ICs was associated with elevated IL-5 secretion in 4/5 donors although the change in IL-5 did not reach statistical significance in 3/5 donors.

### IgG1 Immune Complexes Inhibit Effector Molecule Production by Cytotoxic T Cells

IgG1-ICs did not significantly inhibit the proliferation of memory T cells isolated from peripheral blood of healthy donors. However, the extensive heterogeneity of memory phenotypes precludes categorically concluding that ICs do not directly influence the proliferation of all memory T cells. For instance, negative selection kits we employed do not distinguish central (Tcm) and effector memory (Tem) T cells (**Figure S2**). Tcm and Tem cells possess a higher and low proliferation capacity (44–46), respectively, and diverge in their proportion in peripheral blood across donors. Since Tcm cells express lymphoid-homing receptors (e.g. CCR7), circulating memory T cells tend to be effector-like with naturally lower proliferation capacity and higher cytotoxic propensity (47). Consistently, even in negative controls lacking IgG1-ICs, 50-60% of purified memory T cells from peripheral blood did not divide upon activation, ~2-fold more than their naïve counterparts (53% ± 16% and 56% ± 8% undivided memory *vs.* 22% ± 9% and 20% ± 8% undivided naïve for CD4+ and CD8+ T cells, respectively; p-value <0.0001; **Figures S3B, C**).

CCR7- T cells which include Tem, CD45RA+ terminal effector memory, and their transitional subsets (45, 46) were enriched by sorting (**Figure S2F**). We found that incubation with IgG1-ICs markedly attenuated granzyme-β (Gzmβ) and perforin (Prf) production in an IC-concentration-dependent manner. Specifically, incubation with 40 ug/ml of IgG1 ICs inhibited Gzmβ and Prf to levels comparable to those observed in cultured T cells that had not been stimulated with anti-CD3/anti-CD28 beads (**Figures 3D, E** **)**. No such decrease in Gzmβ or Prf was observed when CCR7- T cells were incubated with either control immune complexes or with monomeric IgG1.

### IgG1 ICs Inhibit Naïve T Cell Differentiation and Induce Hyporesponsiveness but Stimulate a Subset of Memory Progenitors

**Figure 4:**
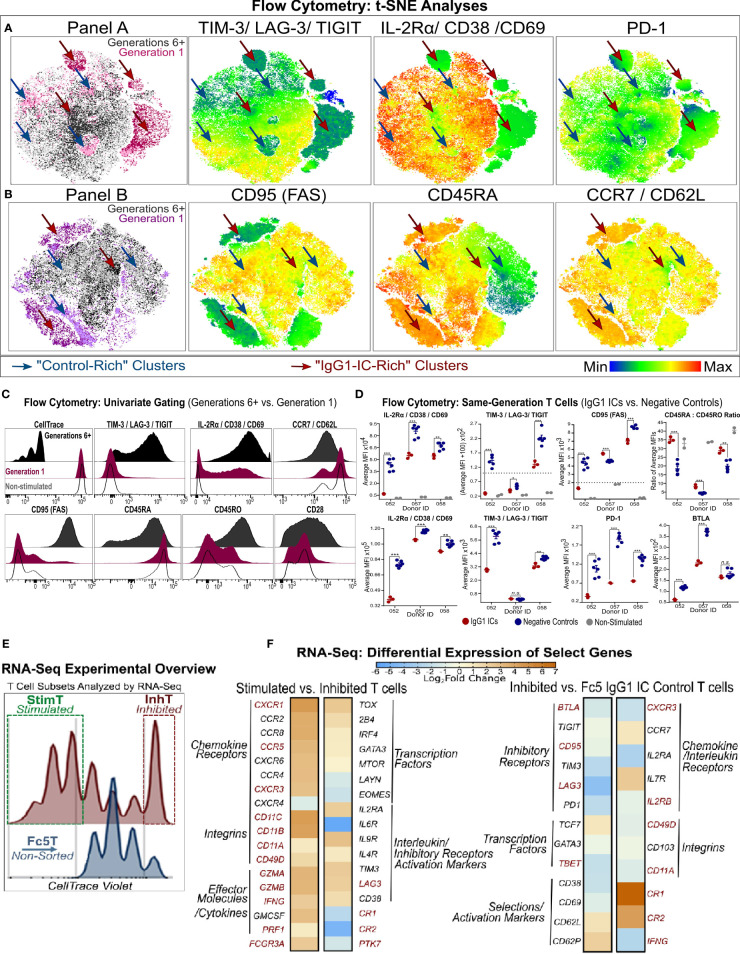
Phenotypic Analysis of Inhibited and Stimulated T cell subsets Following Incubation with IgG1-ICs **(A, B)** t-distributed Stochastic Neighbor Embedding (tSNE) analysis of multi-color flow cytometry panels of activated naïve T cells incubated with IgG1-ICs or controls (Fc5 IgG1-ICs, monomeric IgG1, or TNP-BSA only). Naïve T cells were sampled equally across all controls and donors (N = 3 donors; 2 naïve CD8+ and 1 naïve CD4+). Red and blue arrows are visual aids pointing to “IgG1-IC-rich” or “control-rich” clusters, respectively. Gen1 and Gen6+ populations represent T cells that have not divided or divided at least 5 times, respectively. Gen1 T cells from samples incubated with or without IgG1-ICs are shown in dark and light pink/purple (Panel A/B), respectively. Gen6+ T cells from samples incubated with or without IgG1-ICs are dark and light grey, respectively. Heat-map t-SNE plots are shown for (i) TIGIT, TIM-3, and LAG-3 combination (i.e. TIGIT/TIM3/LAG3 lump gate) (ii) CD25, CD38, and CD69 (i.e. CD25/CD38/CD69 lump gate) and (iii) PD-1 stain. (iv) CD95 (v) CD45RA and (vi) CCR7 and CD62L (i.e. CCR7/CD62L lump gate). Staining for other markers and/or non-stimulated naïve T cells is shown in **Figure S8**. **(C)** Univariate plots for select markers from **(A, B)** are shown. The black and fuchsia histograms represent Gen6+ and Gen1 T cells regardless of treatment (IgG1-ICs and controls). Unfilled histograms pertain to cultured, non-stimulated T cells. **(D)** Average MFI (median fluorescence intensity) is shown specifically for Gen1 (top panels) and Gen6+ (bottom panels) T cells incubated either with IgG1-ICs (red) or controls (blue). Error bars represent the standard error of the mean (SEM). Asterisks ^*^, ^**^, and ^***^ indicate p-values < 0.05, p-values < 0.01, and p-values < 0.0001, respectively. Values below the dashed lines are negative (common, unavoidable consequence of compensation) **(E)** Schematic overview of T cell populations analyzed by RNA-Seq (activated naïve CD8+ T cells incubated with either IgG1-ICs or Fc5 IgG1-ICs). IgG1 IC-treated T cells were sorted for cells stimulated by IgG1-ICs (StimT) and those inhibited by IgG1-ICs (InhT). Fc5 IgG1-IC-treated T cells (Fc5T) are not sorted. **(F)** Differential expression of select genes across StimT, InhT, and Fc5T. Genes are differentially expressed if the adjusted p-value < 0.05 and fold change (FC) ≥ 1.5 in either direction. Select DEGs mentioned in-text are in red. Other genes discussed in-text are shown in **Table S4**.

It is recognized that CD45RA+ CCR7+ naïve-like T cells (43, 45, 48) are heterogenous, comprising multiple subpopulations that exhibit phenotypic and functional differences (49–51). To delineate the effects of IgG1-ICs on phenotypically distinct naïve T cell subpopulations, we utilized flow cytometry to analyze 21 surface markers (**Table S3**) associated with T cell activation, differentiation, and/or dysfunction (e.g. anergy/hyporesponsiveness or exhaustion) (46, 52–54). For this analysis, markers that are typically co-expressed or describe similar phenomena were lumped into one fluorophore channel (e.g. CCR7/CD62L for differentiation; IL-2Rα/CD69/CD38 for activation; TIGIT/TIM-3/LAG-3 for activation/dysfunction). CellTrace Violet was used to distinguish undivided naïve T cells (Generation1; Gen1) and proliferating progeny. We focused on highly proliferative cells that had divided at least five times (Gen6+) (**Figure S3A**). To rule out the involvement of non-conventional T cells (e.g. invariant NKT cells or γδ T cells), purified naïve T cells were confirmed to be TCRαβ+ TCRγδ- CD56- (**Figure S7**). Mucosal-associated invariant T cells (MAITs) were also ruled out, as MAITs present a memory-like phenotype (CD45RA- CCR7-) distinct from naïve T cells (55).

t-SNE analysis (50, 56) revealed that naïve T cell subsets incubated with IgG1-ICs are characterized by differential expression of activation (IL-2Rα/CD38/CD69), immunomodulatory (TIM-3/LAG-3/TIGIT), and certain memory (CD95, CD45RA, CD45RO, CCR7/CD62L) markers (**Figures 4A, B** and **S8A–C**). Compared to highly proliferative cells (Gen6+), undivided (Gen1) T cells in both IgG1-ICs and control samples, appeared to be hyporesponsive and closely mirrored non-stimulated naïve T cells demonstrating: (i) low TIM-3/LAG-3/TIGIT (ii) weak, non-uniform IL-2Rα/CD69/CD38 (iii) high CCR7/CD62L and CD45RA (iv) low CD95 and CD45RO and (v) intermediate CD28 expression (**Figure 4C**). The surface marker profile (i-v) of Gen1 cells is characteristic of naïve and naïve-like memory T cell progenitors (49, 50, 57) demonstrating that even after incubation with anti-CD3/anti-CD28 beads and culture for 5-7 days with IL-2, these undivided, viable T cells retained their naïve-like phenotype.

Undivided Gen1 T cells incubated with IgG1-ICs had (i) lower IL-2Rα/CD38/CD69, TIM-3/LAG-3/TIGIT, and CD95 expression; and (ii) higher CD45RA : CD45RO expression ratio compared to Gen1 T cells in control samples (**Figure 4D**). Similarly, Gen6+ T cells from samples incubated with IgG1-ICs had lower expression of IL-2Rα/CD38/CD69, TIM-3/LAG-3/TIGIT, PD-1, BTLA and CD45RA : CD45RO compared to Gen6+ cells from control samples (**Figures 4D** and **S8D**). These results indicate that IgG1-ICs suppressed the activation and differentiation of naïve T cells regardless of whether or not they had divided.

RNA-sequencing (RNA-Seq) was employed to characterize the transcriptional changes elicited by incubating naïve T cells with IgG1 ICs. Activated naïve CD8+ T cells incubated with IgG1-ICs were first sorted to distinguish inhibited (undivided) naïve T cells from the stimulated subset (referred to as “InhT” and “StimT”, respectively) (**Figure 4E**). We also determined the transcriptional profile of naïve T cells incubated with Fc5 IgG1-ICs (abbreviated as “Fc5T”) which do not affect proliferation (**Figure 2**). DESeq2 was used to analyze differentially expressed genes (DEGs); genes were considered differentially expressed if the adjusted p-value < 0.05 and fold change (FC) is ≥ 1.5 in either direction. Non-DEGs were considered upregulated/downregulated if either the adjusted or nominal p-value < 0.10. p-values for genes discussed herein are specified in **Table S4**. Genes that are discussed below averaged ≥1.2 transcripts per million (TPM) in at least one of the compared subsets (**Table S5**); that is, basal expression (TPM ≥1) of discussed genes was established in InhT, StimT, and/or Fc5T.

CCR7+ CD45+ T cells encompass at least three subsets with a “naïve-like” phenotype (49): (a) recent thymic emigrants (RTEs), the least differentiated subset, reportedly express CD103, PTK7, and markers associated with innate immunity (e.g. CR1, CR2, and IL-8) (58, 59); (b) stem cell memory T cells (Tscm) expressing CD95, IL-2Rβ, CD58 and markers characteristic of effector/memory T cells (e.g. CXCR3, CD11a/b/c) (51, 60); and (c) memory T cells with a naïve phenotype (Tmnp) that differ from Tscm in CD95, CD11a, and IL-2Rβ but express relatively high levels of CXCR3, CD49d, T-BET, and IFN-γ (50, 61). Compared to StimT and Fc5T, InhT cells expressed more CR1, CR2, and PTK7 and less CD95, IL2RB, BTLA, LAG3, TBET, GZMB, IFNG, CD11A/B/C (**Figure 4F** and **Table S4**). Compared to InhT and Fc5T, StimT cells expressed more CXCR3, CD49D, FCGR3A, GZMA, CD11B, CD11C (**Figure 4F** and **Table S4**). StimT cells also differentially expressed higher levels of CXCR1, CCR5, IFNG, GZMB, PRF1, CD11A, and LAG3 compared to InhT cells (**Figure 4F**). While the examined transcriptional features pertain to the final state of activated T cells that had been incubated with IgG1 ICs, inferences can be made about the originating state of the naïve-like T cells; namely, the aforementioned characteristics indicate that naïve T cells whose proliferation is inhibited by IgG ICs are relatively the least differentiated subset presenting immature features that resemble those of RTEs. Conversely, naïve T cells stimulated by IgG ICs have transcriptional features indicative of more differentiated, early memory progenitors with Tmnp-like features.

**Figure 5:**
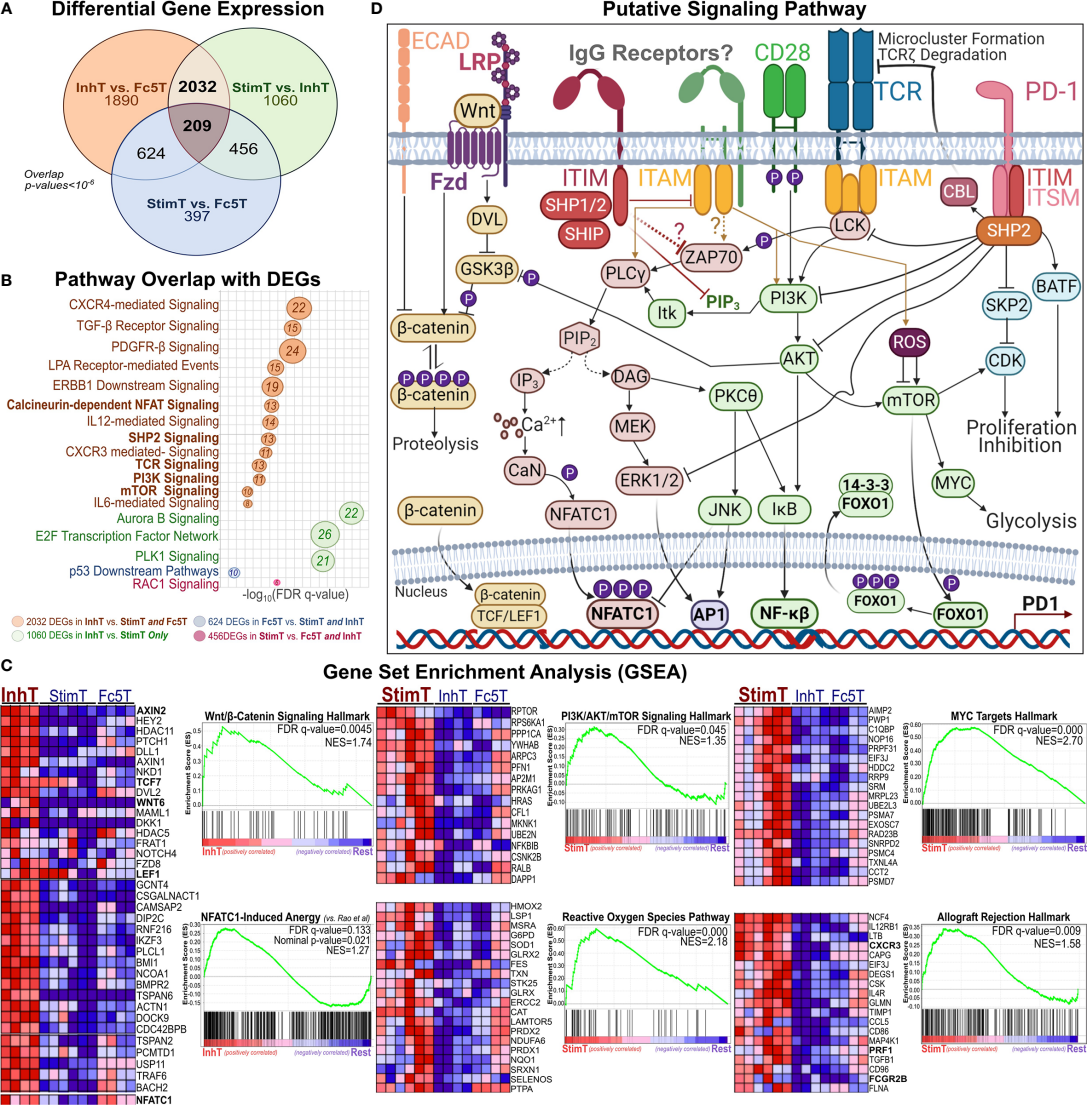
Pathways Perturbed by IgG1 ICs Implicate SHP2, β-Catenin, and AKT/mTOR Signaling **(A)** Venn diagram showing the overlap of differentially expressed genes (DEGs) among StimT, InhT, and Fc5T. Genes are differentially expressed if the adjusted p-value < 0.05 and fold change ≥ 1.5 in either direction **(B)** bubble plot listing select pathways overlapping with observed DEGs. Bubble size is proportional to overlapping number of DEGs. x-axis represents -log(FDR-adjusted q-value) **(C)** Gene set enrichment analysis (GSEA); “Rest” represents any two of InhT, Fc5T, or StimT. For example, “InhT *vs.* Rest” compares InhT to both StimT and Fc5T. The y-axis shows the enrichment score (ES) for members of the gene set. The top portion of the plot shows the running ES for the gene set as the analysis walks down the ranked list of genes. The middle portion of the plot shows where the members of the gene set appear in the ranked list of genes. FDR q-value and the normalized enrichment score (NES) are shown. As recommended by GSEA for non-pre-ranked RNA-Seq, gene sets are considered significantly enriched if the FDR-q value < 0.25. Heatmaps represent select genes contributing to enrichment. By default, GSEA expression values are represented as a range of colors, where red, pink, light blue, and dark blue represent high, moderate, low, lowest expression, respectively. Other enriched pathways are shown in **Table S6** **(D)** Putative signaling network hypothesizing how an IgG1 receptor may theoretically perturb other established T cell pathways.

To elucidate pathways that may be involved in mediating IgG1-IC effects, DEGs were analyzed for overlap with pathways deposited in the MSigDb database (34); and Gene Set Enrichment Analysis (62) (GSEA) was performed to identify transcriptional signatures enriched in each subset. InhT cells differentially expressed 2032 genes (**Figure 5A**) which distinguish naïve T cells inhibited by IgG1-ICs from (a) those stimulated by IgG1-ICs (StimT) and (b) naïve T cells incubated with a control of no/residual IgG-Fc-binding (Fc5T). These DEGs significantly overlapped (FDR q-value < 0.005) with gene sets associated with TCR-, SHP2-, PI3K-, mTOR-, NFAT-, MYC-, and FOXO- signaling (**Figure 5B**). InhT cells also expressed lower levels of PKCθ, NFKB1, and BCL2L1 which are major effectors downstream of CD28 signaling (63) (**Table S4**). Additionally, GSEA revealed that InhT cells were enriched for (a) transcriptional hallmarks of Wnt/β-Catenin signaling (FDR q-value <0.01) and (b) genes known to be induced in dysfunctional T cells that constitutively express an active form of NFAT (35) (FDR q-value <0.135) (**Figure 5C**). Compared to control (Fc5T), InhT cells also expressed more transcripts for TCF7 and LEF1, major recruits of β-Catenin (**Table S4**). Collectively, these results suggest that IgG1-ICs may interfere with CD28 co-stimulation and influence Wnt/β-Catenin signaling.

Compared to StimT and Fc5T, InhT cells expressed more transcripts for FOXO1 and EOMES, which are reported to promote memory formation/survival and prevent anergy (64, 65). Compared to control (Fc5T), InhT cells also differentially expressed more TSC1, which stringently controls mTORC1 activity and maintains naïve T cell quiescence (66) (**Table S4**). StimT cells were enriched for hallmarks of PI3K/AKT/mTOR-, Reactive Oxygen Species (ROS)-, and MYC- signaling (**Figure 5C**); and oxidative phosphorylation. Conversely, Fc5 T cells were enriched for hallmarks of glycolysis, mTORC1-, and E-cadherin- signaling (**Table S6**). Finally, to help visualize how an IgG1 receptor can theoretically regulate T cell function, the aforementioned well-established pathways (66–70) are shown together in **Figure 5D**.

### T Cells Express Multiple Antibody Receptors Including IgG Receptors

**Figure 6:**
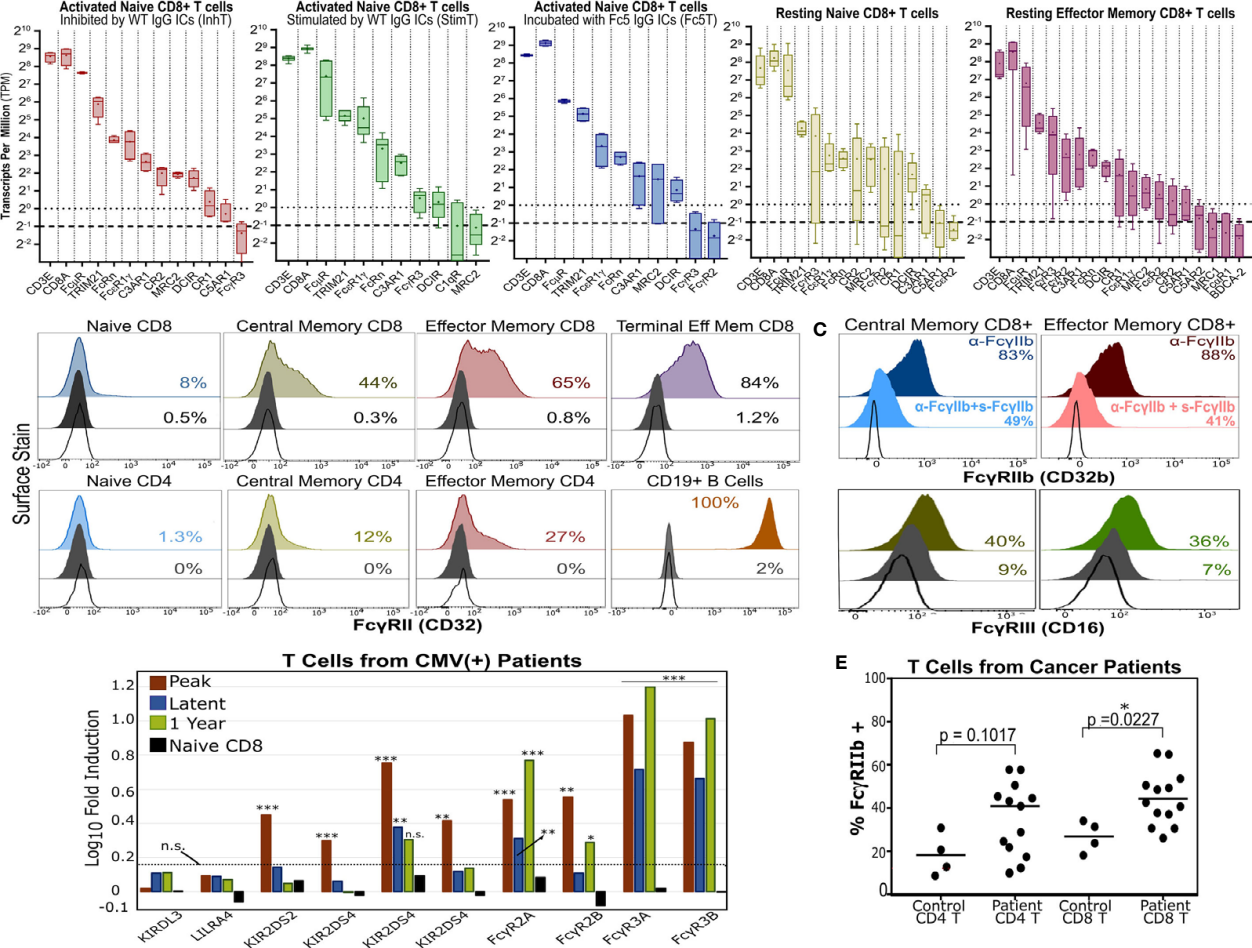
T Cells Express Multiple Antibody Receptors. **(A)** mRNA of lectins and several antibody receptors in various T cell subsets. Results for “resting” subsets derive from RNA-Seq data published by Ranzani et al. (48) and pertain to sorted, non-stimulated cells (4-5 donors). The median (line), mean (dot), minimum/maximum(whiskers), and interquartile range (box) are shown. The dotted and dashed lines represent the TPM=1 (expressed) and TPM=0.5 (very low expression) thresholds. TPMs for CD3E and CD8A are shown for reference. **(B)** Surface expression of FcγRII (CD32) in various unstimulated T cell subsets. B cells serve as a positive control. **(C)** FcγRIIb or FcγRIII surface expression in memory CD8+ T cells. For FcγRIIb, T cells were stained with an aglycosylated FcγRIIb antibody with or without competing soluble, non fluorescent FcγRIIb. **(B, C)** Dark gray and unfilled histograms represent isotype controls and non stained cells, respectively. Displayed percentages correspond to % positive events set relative to unstained cells. **(D)** Differential FcγR gene expression in CD8+ T cells of HCMV-infected patients (vs. healthy donor CD8+ T cells). Results derive from microarray RNA data published by Van Stijn et al. (39). Various timepoints post-infection are shown (3 patients; 3 healthy donors). Non-FcγR genes considered differentially expressed or unchanged by Van Stijn et al. are shown. Asterisks *, **, and *** represent p-values <0.05, p-values <0.01, and p-values < 0.001, respectively. n.s. stands for statistically not significant. The dashed line denotes the fold induction threshold below which no statistical significance was observed. **(E)** FcγRIIb display in T cells isolated from PBMCs of healthy donors or cancer patients. The percent of FcγRIIb(+) events is determined based on the FMO gate.

We endeavored to survey various T cell subsets for the expression of antibody receptors that could potentially link T cell and B cell immunity *via* ICs. Whereas FcγRs are canonical receptors that exclusively bind IgG Fc, non-canonical FcRs (nc-FcRs) can bind other antibody classes and include C-type lectins (CLEC), Fc-receptor-like molecules (FcRLs), and mannose receptors (9, 11, 12). We analyzed our RNA-Seq data and the dataset (48) published by Ranzani et al. that analyzed the transcriptome of resting purified T cell and B cell subsets. We searched for all non-pseudogenes that encode lectins (CLEC-, SIGLEC-), glycan receptors (e.g. mannose receptors), or known IgG receptors. For various T cell subsets, we then ranked genes at least 0.25 transcripts per million (TPM) (**Figure S9**) and focused on receptors with reported binding to any antibody class (**Table S7**). InhT, StimT, Fc5T, and/or other resting T cell subsets expressed transcripts for complement receptors (CR1, CR2, C3AR1, C5AR1, C1qR) and various antibody receptors including exclusive IgG-Fc-binders (FcγRII, FcγRIII, FcRn), IgM receptor FcμR, TRIM21, MRC2 (Endo180), DCIR (CLEC4A), and FcϵRII (**Figure 6A**).

Transcriptionally, naïve T cells showed low FcγR expression and high expression of FcμR, MRC2, DCIR, and FcRn (**Figure 6A**). Consistent with mRNA levels (**Figure S9**), human naïve T cells upregulated FcγRII surface expression upon differentiation *in vivo* (**Figures 6B** and **S10**). Since little FcγR protein was observed in resting naïve T cells, we checked if FcγRII/III are potentially upregulated upon activation *in vitro*. Staining with anti-FcγRII and anti-FcγRIII increased gradually over time upon T cell activation *in vitro*; however, isotype staining also increased (despite testing multiple clones) which precluded confidently gauging FcγRII upregulation and distinguishing it from Fc-binding by nc-FcRs (**Figure S11**). Memory CD8+ T cells expressed the highest levels of FcγR mRNA and specifically expressed FcγRIII and inhibitory FcγRIIb on the cell surface (**Figure 6C**).

Interestingly, antibody receptors were upregulated in T cells from patients with certain disorders. For example, analyzing microarray data published by Van Stijn et al. (39) revealed that CD8+ T cells from CMV-infected patients differentially expressed more FCGRII/III compared to healthy donors (**Figure 6D**). T cells isolated from PBMCs of various cancer patients (**Table S8**) upregulated inhibitory FcγRIIb surface expression (**Figure 6E**). We further analyzed other published RNA-Seq datasets from healthy donors and hepatocellular carcinoma patients and observed similar trends (**Figure S12**). Moreover, FcγRIIb/III expression was also observed in murine naïve T cells and OT-1 and OT-2 T cells activated *in vitro* and in a melanoma tumor model (**Figure S13**). These results (a) support recent reports (20, 24, 25) demonstrating FcγRII/III expression in memory T cells; (b) show that FcγR expression can be further upregulated in disease settings like cancer and viral infections; and (c) demonstrate that a direct link between T cell and B cell immunity as mediated by potential T cell expression of antibody receptors should not be ignored.

**Figure 7:**
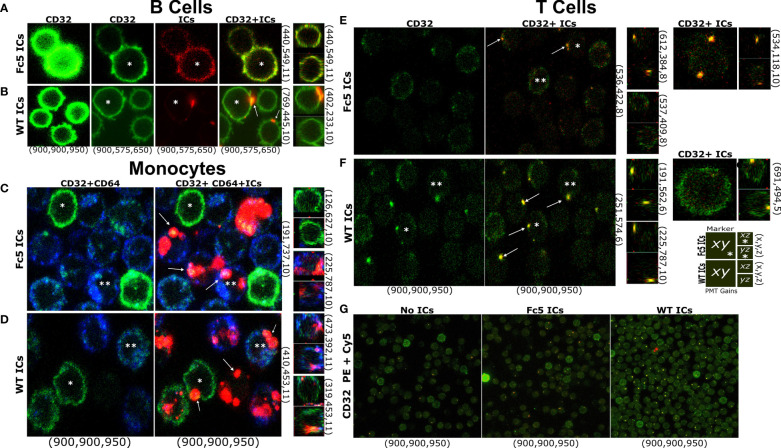
T Cells Bind IgG Immune Complexes in a Manner Similar to B cells and Monocytes **(A–G)** Confocal microscopy of FcγR display and IC staining in total T cells, B cells, or monocytes. **(A–F)** Left row labels indicate fluorescent ICs (WT- or Fc5- IgG1-ICs). Top column labels refer to single-channel or merged images (FcγRII (CD32): green; ICs: red; FcγRI (CD64): blue). Bottom column labels indicate PMT gains used for each detector (PE: green, Cy5: red, BV421: blue-in this order) during image acquisition. Right row labels indicate the x-y-z plane coordinates. Larger and smaller images within each panel correspond to the x-y or x-z/y-z planes, respectively. A schematic legend summarizing the employed labelling system is shown. Asterisks help track the same cell in different images across a row. Arrows emphasize detected fluorescent clusters. For **(G)** top and bottom labels indicate IC type and PMT gains, respectively.

Finally, while our data demonstrates that resting T cells do not express FcγRs at levels that are comparable to B cells or monocytes, we used confocal microscopy to examine whether IgG1-ICs can bind T cells (total CD3+ TCRαβ+ cells) in a manner similar to B cells and monocytes which express exclusively high levels of FcγRIIb and all FcγRs, respectively (5, 8) (e.g., IC stains were on the membrane and not internalized; the profile of staining clusters, etc.). Cells were stained with lineage markers and fluorescent WT- or Fc5- IgG1-ICs. At the same photomultiplier (PMT) gains as T cells and monocytes, B cells were the brightest for FcγRII staining; thus, to avoid signal saturation, PMT gains were adjusted down for B cells (**Figures 7A, B** **)**. Due to their FcγR profile, B cells were expected to bind WT- but not Fc5- IgG1-ICs, whereas monocytes should bind both. Expectedly, WT IgG1-ICs bound B cells. Also, unlike the binding profile of Fc5 IgG-ICs, which was more uniform resembling background staining or residual binding, WT IgG1-IC-stains appeared in concentrated clusters and localized with FcγRII stains (**Figures 7A, B** **)**. Conversely, monocytes had similar binding profiles for Fc5- and WT- IgG1-ICs; and Fc5 IgG1-IC-clusters localized with FcγRI stains (**Figures 7C, D** **)**. Interestingly, like the staining profile (albeit not intensity) of B cells, T cells had a clear presence of clusters when incubated with fluorescent WT IgG1-ICs, which also localized with FcγRII stains (**Figure 7F**). While a few spots were also discernible with T cells stained with fluorescent Fc5 IgG1-ICs (**Figure 7E**), these stains were similar in level to T cells that were not incubated with fluorescent ICs and were outnumbered by T cells stained with WT IgG1-ICs under the same conditions (**Figure 7G**; ~33.5 *vs* 15 “yellow” IC-FcγII clusters per 100 cells for WT- *vs.* Fc5- IgG ICs, respectively).

## Discussion

An expanding body of evidence especially in recent years has challenged the early “canonical” view that T cells do not express antibody receptors (20–25). Interestingly, circulating immune complex (CIC) levels have been reported to be higher in some cancer patients compared to healthy individuals and have been used as negative prognosticators (4, 71, 72). While these correlations do not establish causation, they raise an interesting question; namely, can CICs inhibit immune responses?

Here we examined the phenotypic consequences of well-defined soluble ICs on naïve and memory T cell function. We found that IgG1-ICs, but not control ICs formed by antibodies of different isotypes or by aglycosylated IgG1 antibodies that only bind to Fc*γ*RI (and FcRn) but not Fc*γ*RII/III (a) inhibited naïve T cell proliferation and differentiation; but (b) also stimulated the division of a subset of naïve-like progeny. Phenotypic and transcriptional profiling analyses suggested that ICs inhibit the proliferation of a subset of naïve T cells that resemble recent thymic emigrants (49). Conversely, the stimulated subset resembled early memory progenitor T cells with a naïve-like phenotype. IgG1-ICs did not affect the proliferation of memory T cells from peripheral blood of healthy donors (57). However, we show that IgG1-ICs suppressed the production of granzyme-β and perforin in human effector memory T cells, a finding that is consistent with observations in mice (20). Further, our RNA-Seq analyses implicated SHP2-, Wnt/β-Catenin-, and PI3K/AKT/mTOR- signaling pathways in the mediation of IgG1-IC effects on naïve T cells.

To proliferate and acquire effector functions upon TCR stimulation, T cells require the cooperation of transcription factors NFAT, NF-κβ, and AP-1. Upon TCR engagement, Lck phosphorylates ITAMs of CD3/TCRζ chains leading to Zap-70 activation and, ultimately, phospho-lipase C-γ (PLC-γ) recruitment. To effectively activate PLC-γ, naïve T cells require co-stimulation (e.g. *via* CD28) which activates NF-κβ *via* the PI3K-AKT-PKCθ-CARMA1 axis (73, 74). Multiple inhibitory receptors (e.g. PD-1, CTLA-4, and TIGIT) interfere with TCR and/or CD28 signaling. Via their ITIM, ITSM, and/or other motifs, these receptors recruit phosphatases, like SHP-1/2 and/or SHIP-1, which can suppress kinases and inhibit mTOR or NF-κβ activation (68, 75). Our RNA-Seq results suggest that IgG1-ICs may influence T cell function by interfering with CD28 co-stimulation (speculatively, *via* the recruitment of SHP and Wnt/β-Catenin signaling without shutting off NFAT-signaling triggered by TCR stimulation). Defective CD28 signaling can impair NF-κβ activation and reduce PKC-θ activity, which is necessary for AP-1 activation and induces anergy in CD8+ T cells (76, 77). Also, while NFAT is vital for T cell function when activated in the appropriate context, NFAT activity under suboptimal conditions (e.g. inefficient activation of or cooperation with AP-1) is established to induce T cell anergy/hyporesponsiveness (35). Moreover, downregulation of Wnt/β-Catenin signaling is reportedly required for clonal expansion of CD8+ T cells (70). β-Catenin also inhibits NF-κβ (78); is stabilized by SHP2 (79); and reportedly induces anergy in naïve CD4+ T cells (80). Taken with these reports, our results (a) hint that IgG1-ICs may influence T cell function by binding an IgG1 receptor that leads to the recruitment of phosphatases like SHP1/2; and (b) warrant further studies that can conclusively determine the mechanisms *via* which IgG1-IC signals are transduced. While this proposed mechanism cannot exclude other possibilities (e.g., IgG1-ICs may directly interfere with CD3/TCRζ ITAM signaling by recruiting a phosphatase that directly attenuates TCR ITAM or Zap70 phosphorylation), it can explain why IgG1-ICs (a) did not significantly impair memory T cell proliferation, which do not stringently require CD28 co-stimulation like naïve T cells do; but (b) severely diminished cytotoxic potential which is amplified by CD28 signaling (70, 73).

Finally, we sought to (a) illuminate the expression profile of receptors that could bind antibodies in various T cell subsets/states (memory/disease) and (b) utilize that insight to surmise which IgG1 receptors could theoretically be mediating IgG1-IC effects in naïve and cytotoxic memory T cells. Consistent with recent reports (19, 20, 24, 25), we demonstrated that differentiated memory T cells displayed FcγRIIb and FcγRIII on the cell surface. Memory T cells also expressed transcripts for complement (C3AR1) and other nc-FcRs (FcRn, TRIM21, FcϵRII, and FcμR). While IgG1-IC-stimulated, activated naïve T cells (StimT) differentially expressed more FCGR3A transcripts, activated IgG1-IC-inhibited (InhT), activated control (Fc5T), and resting naïve T cells did not express FCGR transcripts. Instead, InhT cells expressed transcripts for (a) IgG-binding nc-FcRs (FcRn, TRIM21, MRC2, and DCIR); (b) the IgM receptor FcμR; and (c) complement receptors CR1, CR2, C3AR1, and C5AR1. Also, while naïve T cells clearly upregulated FcγRII surface display upon differentiation *in vivo*, resting naïve T cells did not display appreciable FcγR protein, and we were unable to conclusively demonstrate upregulation of FcγRII or FcγRIII surface display upon *in vitro* activation due to a concomitant increase in isotype staining.

Moreover, our results indicated that only WT IgG1-, but not IgA1-, IgE-, or Fc5 IgG1-ICs inhibited naïve T cell proliferation. Accordingly, the involvement of most aforementioned receptors can be excluded by elimination since (a) TRIM21 is an intracellular, stimulatory receptor that binds IgG, IgA, and IgM (9); (b) FcϵRII binds IgE but not human IgG1 (9); (c) IgM and IgG1 [and to a much lesser extent IgA (81)] are potent complement mediators; however, our cultures utilized heat-inactivated fetal bovine serum (Gibco) which is established to lack complement activity (82); (d) FcμR does not bind IgG1 (9); and (e) Fc5-IgG1 binds FcRn comparably to WT IgG1 (42). As for the remaining nc-FcRs, MRC2 reportedly binds agalactosyl-IgG (11) and possibly IgA (83); whereas DCIR binding to IgG is unclear (84). Therefore, while a cooperative or synergistic role cannot be excluded for other receptors discussed in (a)-(e) (e.g., despite the use of heat-inactivated FBS, we cannot completely rule out that some of the reported effects may be, at least in part, mediated by IgG1-ICs *via* complement receptors due to minute quantities of residual fetal bovine serum complement components), FcγRs, MRC2, DCIR, and/or a novel unidentified IgG1 receptor could theoretically be responsible for observed IgG1-IC effects in naïve and/or memory T cells.

In summary, our study clearly demonstrated that IgG1-ICs can profoundly and directly influence naïve and memory T cells, clarifying that both subsets express IgG1 receptors. We also demonstrate that T cells express various receptors such as FcγRs, complement receptors, FcRn, and FcμR that could theoretically link their responses to B cell immunity *via* antibodies in the context of immune complexes. While our studies do not conclusively identify the culprit IgG1 receptor(s), our observed IgG1-IC effects foretell that IgG-ICs, IgM-ICs, and complement may also modulate T cell function *in vivo*. Thus, investigating potential antibody-receptor/IC-mediated clinical effects and employing gene-editing technologies to specifically identify all antibody receptors that may regulate T cells are highly warranted. Understanding how ICs influence T cells (e.g. within solid tumor microenvironments) can guide our therapeutic designs. Further, appreciating T cell-IC-antibody-receptor interactions may illuminate (a) the engineering of therapeutic T cells (e.g. CAR-T cells) and (b) interpretation of clinical outcomes and correlations with patient starting material and disease characteristics (85, 86).

## Data Availability Statement 

The RNA-Seq data generated in this study are deposited in the NCBI repository (https://www.ncbi.nlm.nih.gov/) under the accession number GSE166445.

## Ethics Statement

The studies involving human participants were reviewed and approved by Institutional Review Board (IRB) at the University of Texas at Austin. The patients/participants provided their written informed consent to participate in this study. The animal study was reviewed and approved by Institutional Animal Care and Use Committee (IACUC) at the University of Texas at Austin.

## Author Contributions

WC, MD, and GG conceived and designed the research. WC,MD, MR, JM, SS, DG, KK, and JK performed experiments. WC, HS,and VI analyzed data. WC and GG wrote the paper. All authors contributed to the article and approved the submitted version.

## Funding

This work was supported by grants from the Clayton Foundation, the National Institute of Health (NIH U01AI148118), and The University of Texas at Austin.

## Conflict of Interest

WC is employed by Juno Therapeutics a Bristol-Myers Squibb Company; HS is employed by Genentech; and MD is employed by Incyte Corporation. None of the work reported here is supported by or affiliated with these organizations.

The remaining authors declare that the research was conducted in the absence of any commercial or financial relationships that could be construed as a potential conflict of interest.

## Publisher’s Note

All claims expressed in this article are solely those of the authors and do not necessarily represent those of their affiliated organizations, or those of the publisher, the editors and the reviewers. Any product that may be evaluated in this article, or claim that may be made by its manufacturer, is not guaranteed or endorsed by the publisher.
