# Supplementary material for: IgG Immune Complexes Inhibit Naïve T Cell Proliferation and Suppress Effector Function in Cytotoxic T Cells
Source: Front Immunol. 2021 Aug 10;12:713704. doi: 10.3389/fimmu.2021.713704 (PMC8383740; doi:10.3389/fimmu.2021.713704)
Supplement: Supplementary file 1 [file DataSheet_1.pdf]

Supplementary Information

Supplementary Figures (S1-S13)

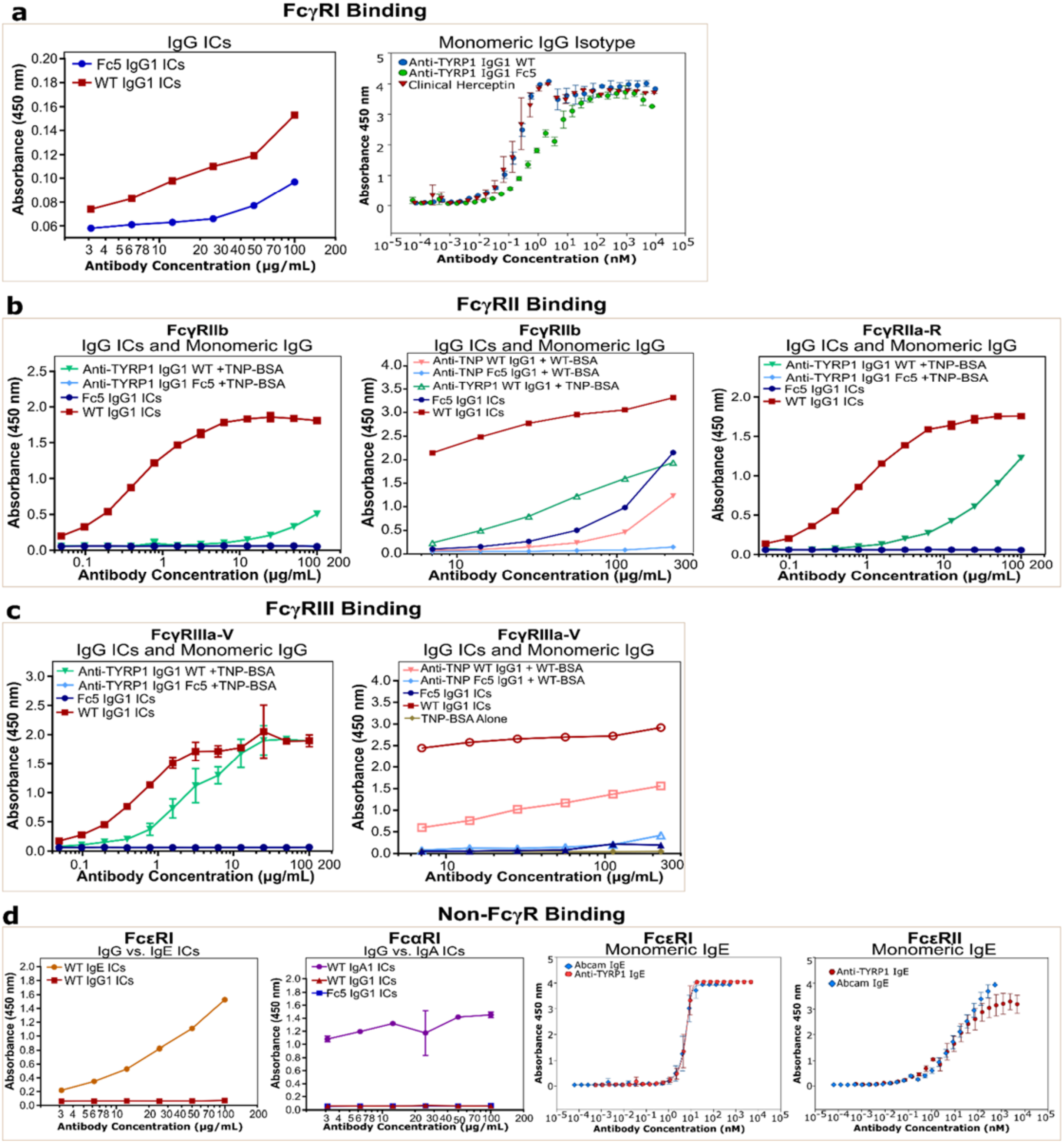

Figure S1 | Only IgG1 ICs, not Monomeric IgG1 or Fc5 IgG1 ICs, can effectively bind low-affinity FcγRs ELISA binding of various immune complexes and monomeric mAb controls to (**a-c**) FcγRs and (**d**) IgA and IgE Fc receptors, FcαRI and FcεRI/II, respectively. As expected, wild-type (WT) IgG1-ICs bound to purified low-affinity FcγRs with EC50s that were >10-100-fold lower relative to monomeric IgG (**b-c**). Monomeric Fc5 IgG1 and Fc5 IgG1-ICs bound only to FcγRI and not to any of the low affinity receptors (**a-c**). Likewise, IgA1-ICs and IgE-ICs, but not IgG1-ICs, bound FcαRI (IgA FcR) and FcεRI (IgE FcR), respectively (**d**).

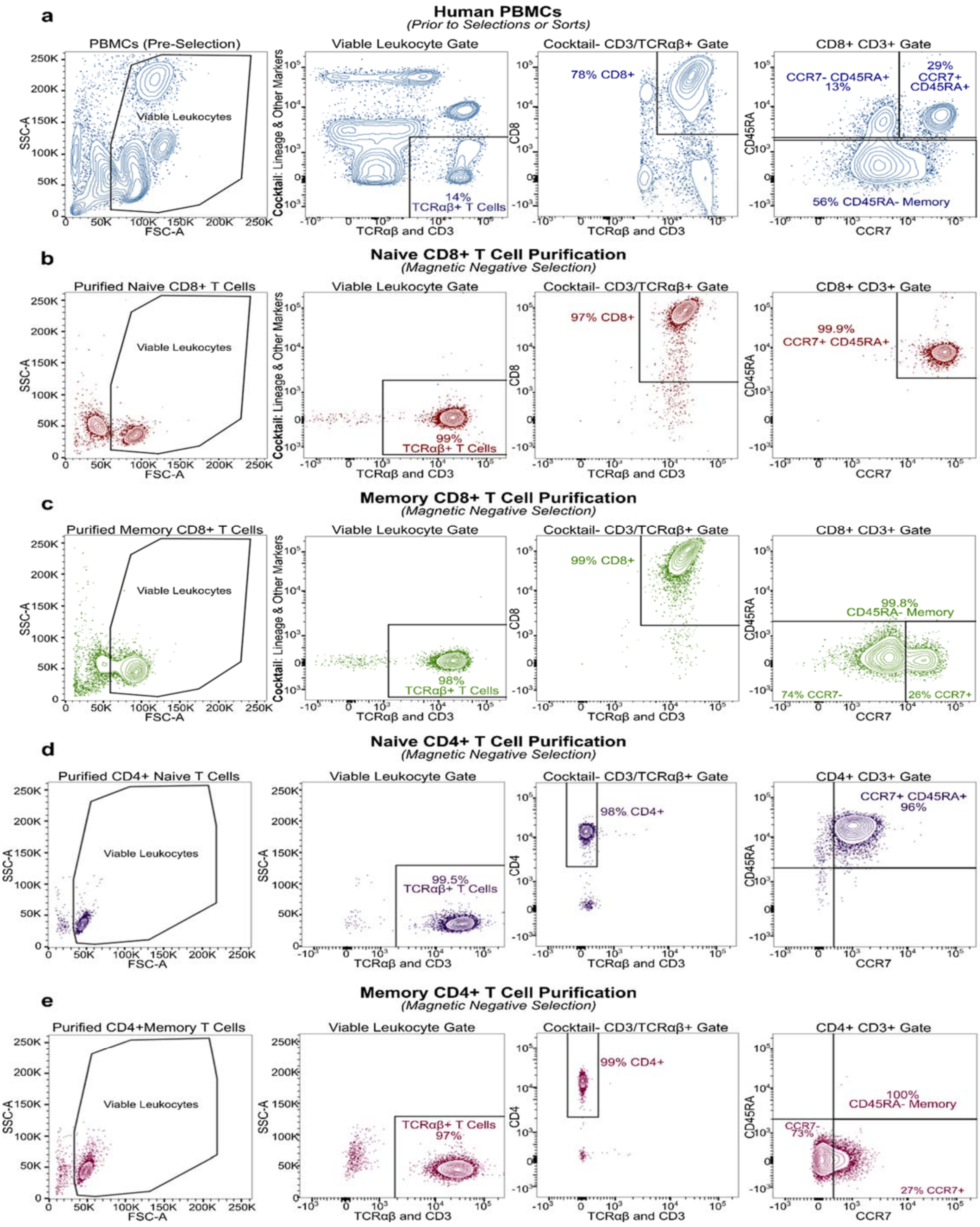

Figure S2 | Naïve and Memory CD4+ or CD8+ T Cells were Negatively Selected from Human Peripheral Blood Mononuclear Cells

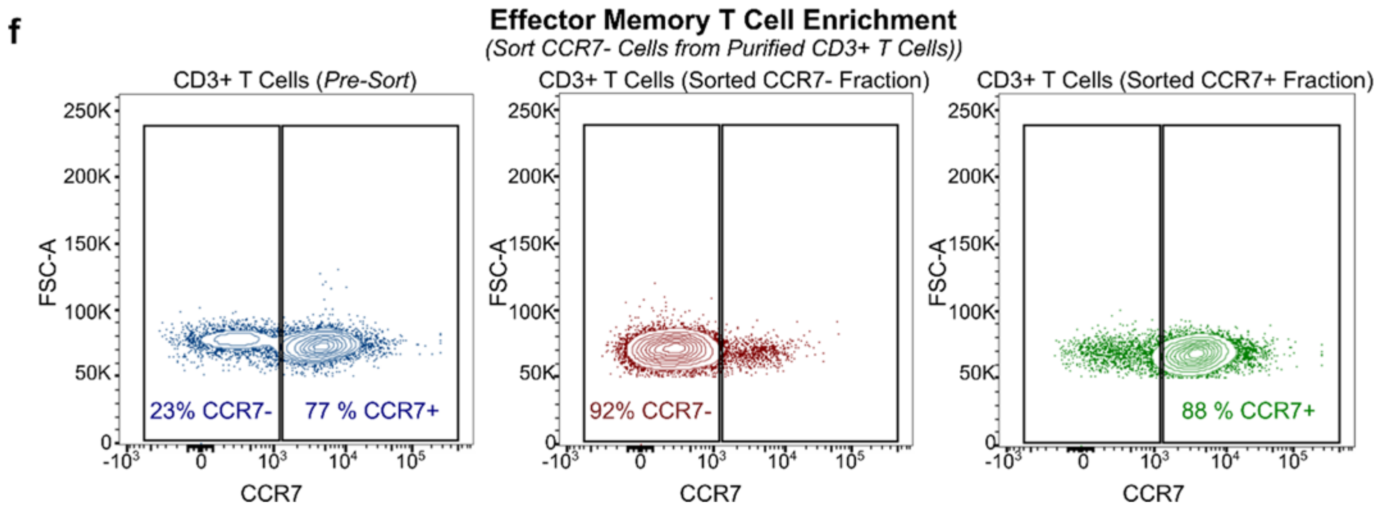

Figure S2 Continued | Naïve and Memory CD4+ or CD8+ T Cells were Negatively Selected from Human Peripheral Blood Mononuclear Cells Representative purity results are shown for the negative selection from (**a**) PBMCs of (**b**) Naïve CD8+ T cells (**c**) Memory CD8+ T cells (**d**) Naïve CD4+ T Cells and (**e**) Memory CD4+ T Cells by magnetic selection kits. (**f**) Representative purity results are shown for the enrichment of CCR7- effector memory T cells from total CD3+ T cells isolated from human PBMCs via flow cytometry sorting.

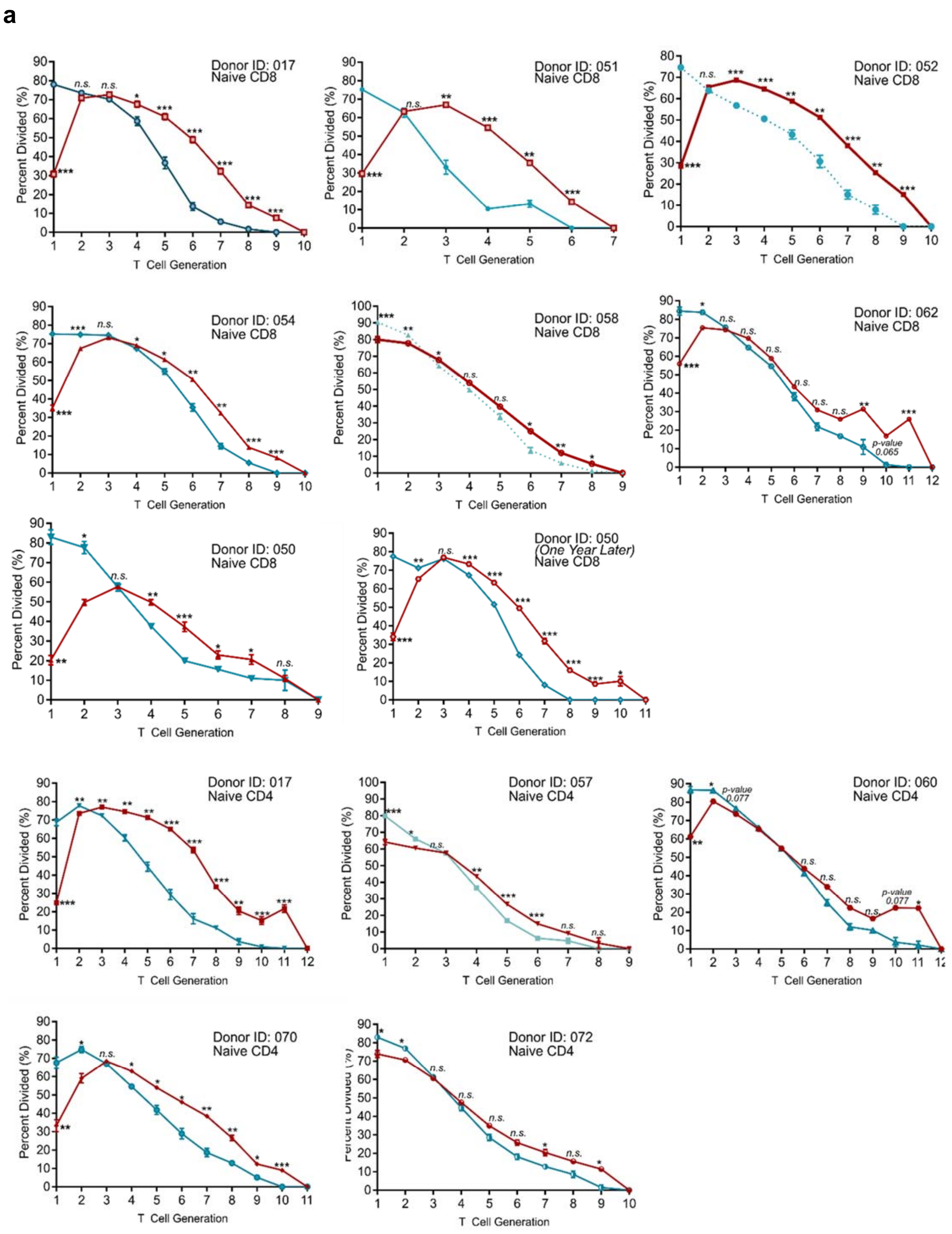

Figure S3 | IgG1-ICs Inhibit Naïve T Cell Proliferation but Stimulate a Subset of Dividing Progeny

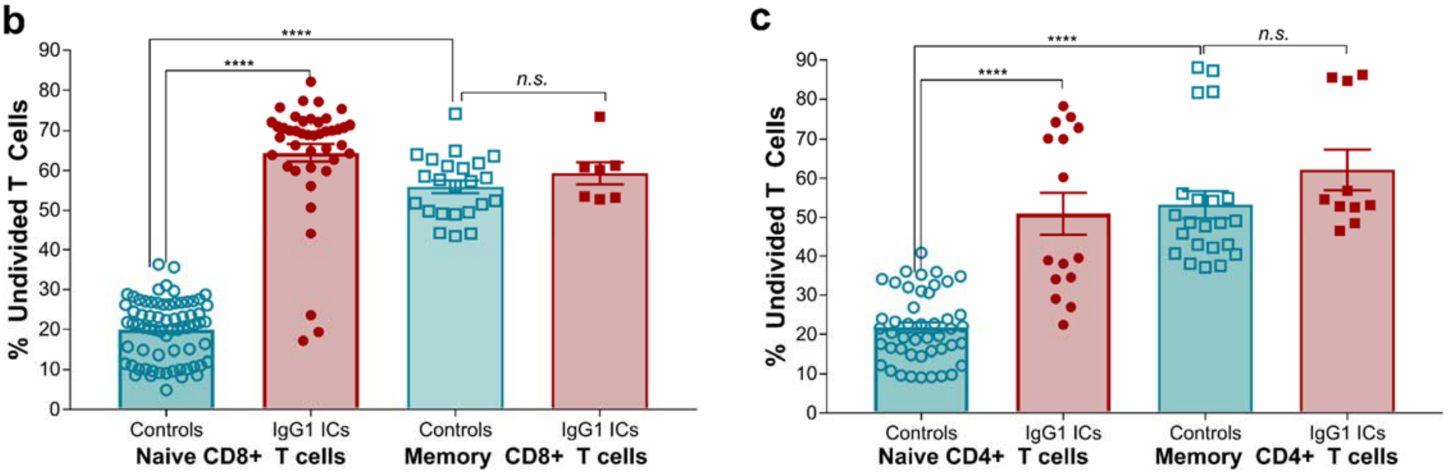

Figure S3 Continued | IgG1-ICs Inhibit Naïve T Cell Proliferation but Stimulate a Subset of Dividing Progeny (**a**) Plots of division (θ_i) vs T cell generation (i) for WT IgG1-ICs (red) or negative controls (blue) show that IgG1-ICs inhibit naïve T cell proliferation (Generation 1) and stimulate a subset of dividing progeny. The latter phenomenon is observed as early as after 2-3 divisions and consistently in later divisions (Generation 6+). Negative controls include Fc5 IgG1-ICs, monomeric IgG1, TNP-BSA only, PBS, and/or other negative controls (**Table S1**). Mean values are plotted. Error bars represent the standard error of the mean (SEM) for each group. p-values are calculated using unpaired two-tailed t-tests adjusting for multiple comparisons using the Sidak-Holm correction. Consistent standard deviation is not assumed unless an experiment pertains to a single-well assay. Asterisks *, **, and *** indicate p-values < 0.05, p-values < 0.01, and p-values < 0.0001 respectively. "n.s." stands for statistically not significant. θ_i (or 100-Φ_i) is % division (or 100-% undivided) for a particular T cell generation (i). For instance, θ_1 is equal to (100- Φ_1) where Φ_1 is % undivided. Φ_1 can be calculated using the following equation:

$$\Phi_1 = \frac{X_1}{X_1+\sum_{i=1}^{D}\frac{X_{i+1}}{2}}$$

where D is the total number of observed divisions (number of peaks -1) and X_1 is the frequency of the 1st peak gated in FlowJo. To calculate Φ_2, the same equation is used but "X_1" would need to be recalculated by excluding generation 1 before gating. To calculate Φ_3, the same equation is used but "X_1" would need to be recalculated by excluding generations 1 and 2 before gating- so on and so forth. A template excel sheet that automatically calculates and plots θ_i (or Φ_i) vs. is provided as a supplemental attachment. Alternatively, % undivided (Φ_1) and other proliferation parameters can be calculated by the proliferation plugin in newer versions of FlowJo.

(b-c) Bar graph of percent undivided T cells (Φ_1) for naïve and memory CD4+ and CD8+ T cells. The graph presents the data aggregate across all independent experiments, donors, and negative controls. p-values were calculated using one-way ANOVA. Asterisks **** indicate p-values < 0.0001. "n.s." stands for statistically not significant.

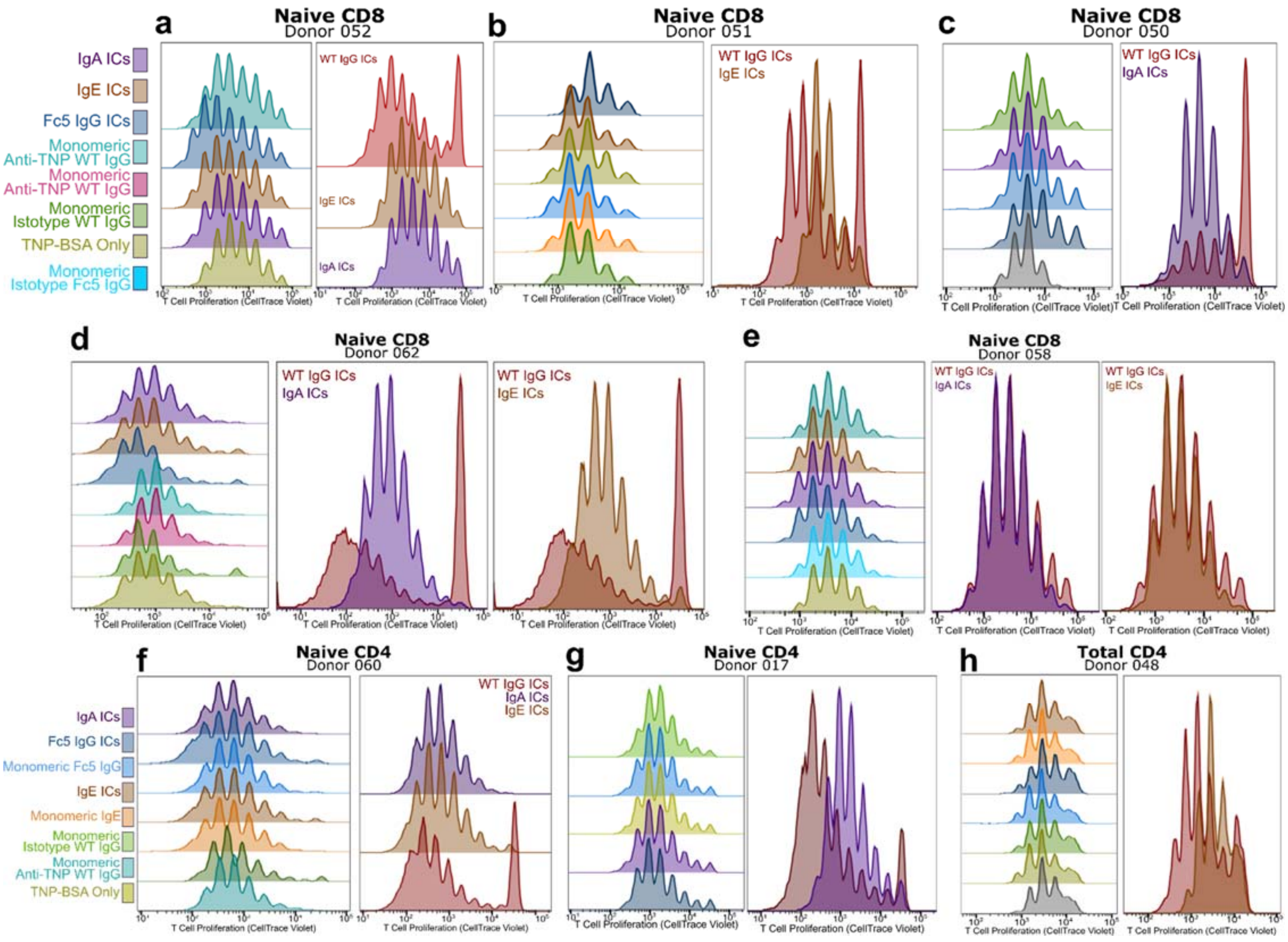

Figure S4 | Only WT IgG Immune Complexes, but not IgA1 or IgE Immune Complexes, Inhibit Naïve T Cell Proliferation Division of CellTrace-stained CD8+ or CD4+ T cells activated with Dynabeads and incubated with WT IgG1-ICs (red), Fc5 IgG1-ICs (dark blue), IgA1-ICs (purple), IgE-ICs (dark orange), or other negative controls. Results for Donors 062 and Donor 060 are also presented in **Figure 2e** and are shown here overlaid with control ICs and next to all the negative controls utilized in these experiments.

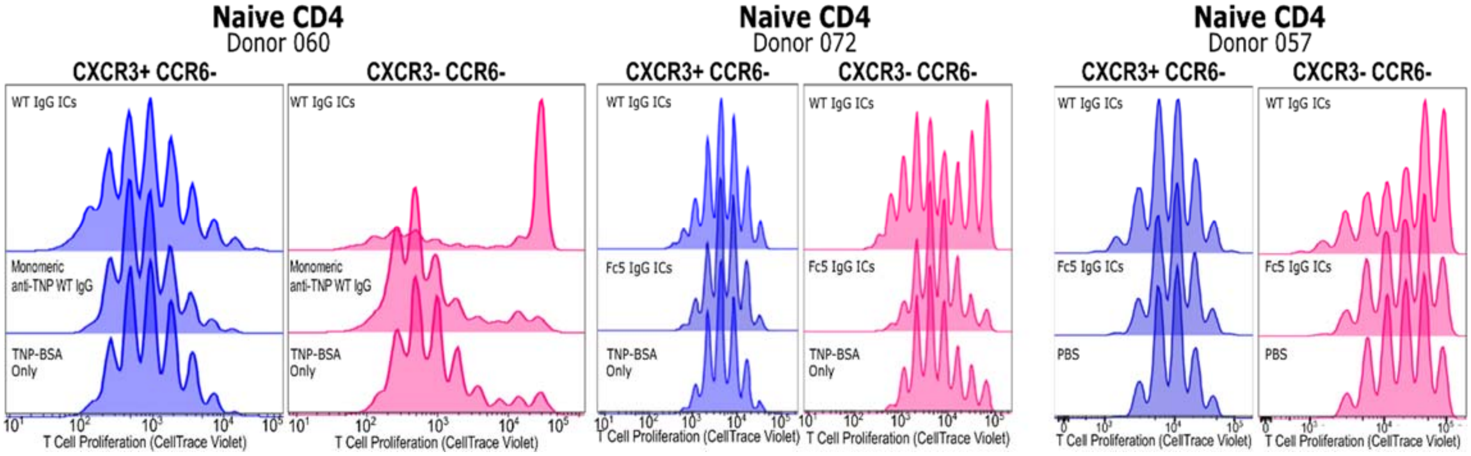

Figure S5 | CCR6- CXCR3- Naïve CD4+ T cells are Profoundly Inhibited by WT IgG Immune Complexes Naïve CD4+ T cells activated with Dynabeads and incubated with IgG1-ICs or negative controls are cultured for 5-7 days and stained with anti-CXCR3 and anti-CCR6 mAbs to examine proliferation of Th1-like (CXCR3+ CCR6-) and Th2-like (CXCR3- CCR6-) phenotypes. Results for Donor 070 are shown in main **Figure 1**.

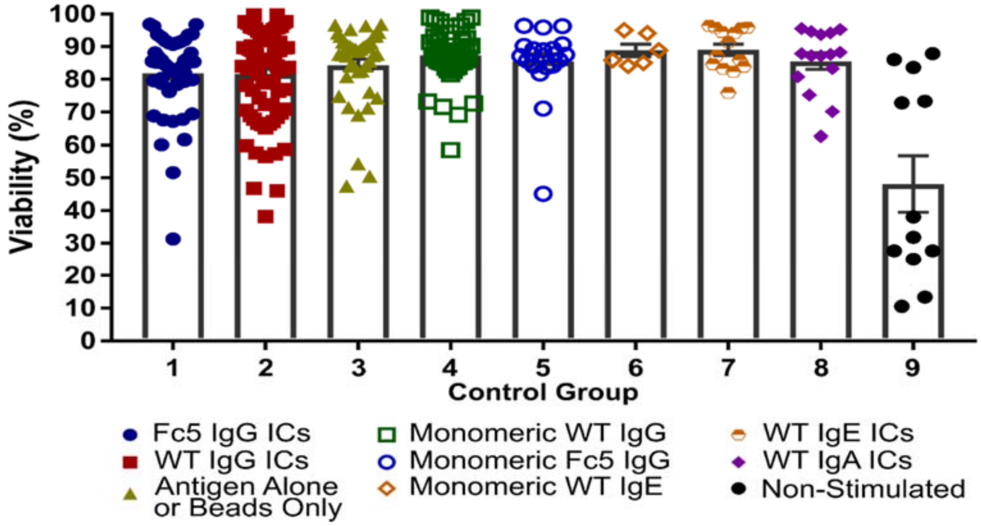

Figure S6 | IgG Immune Complexes Do Not Change Overall Culture Viability The viability of naïve, memory, and total T cell cultures that are either non-stimulated or activated with anti-CD3/anti-CD28 Dynabeads in the presence of various controls (e.g. Fc5 IgG1-ICs, WT IgG1-ICs, or monomeric IgG1). The bars represent the mean and the error bars represent the standard error of the mean (SEM).

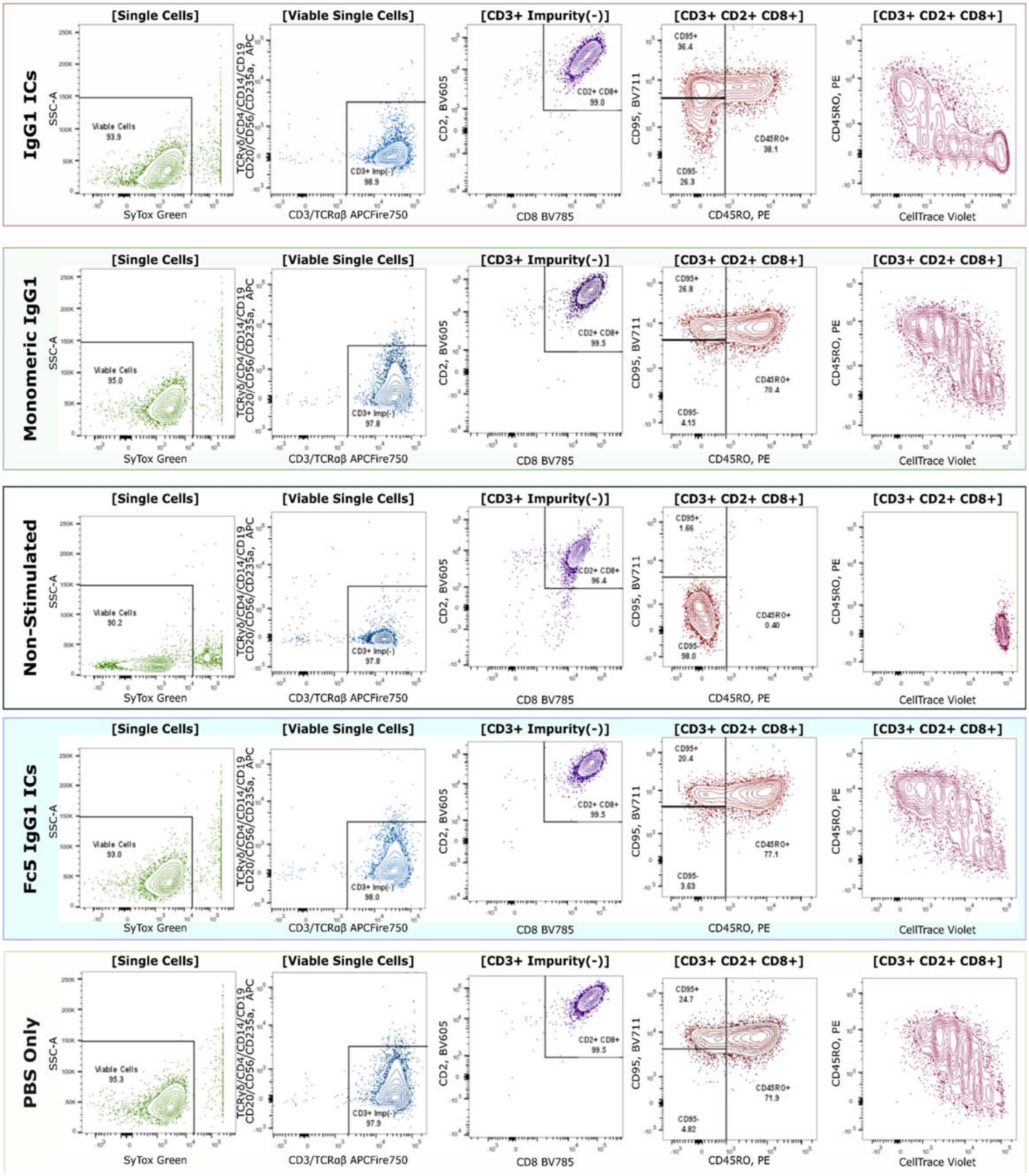

Figure S7 | Naïve T cells Inhibited/Stimulated by WT IgG ICs are TCRαβ+ T cells Flow cytometry plots of naïve CD8+ T cells (Donor 052) activated with anti-CD3/anti-CD28 Dynabeads and incubated with IgG1-ICs or negative controls. T cells were stained with anti-CD2, a cocktail of anti-CD3 and anti-TCRαβ, anti-CD45RO, anti-CD95, viability dye (SyTOX Green), and a cocktail of antibodies targeting CD14, CD19, CD20, CD56, TCRγδ, CD235a, and CD4.

Flow Cytometry: t-SNE Analyses

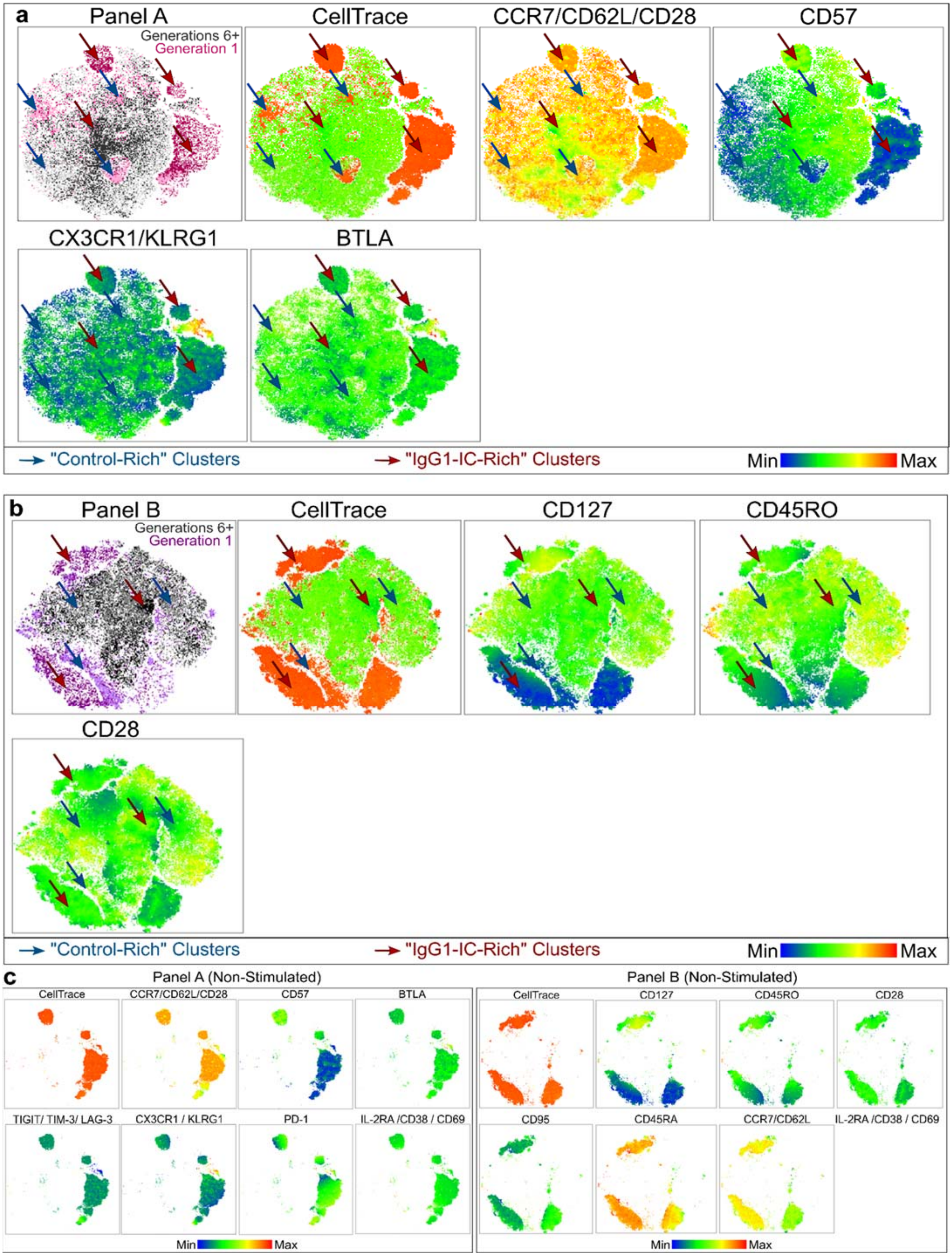

Figure S8 | Other Markers Included in t-SNE Analyses and Gene Expression Data Complementing t-SNE Analyses

d

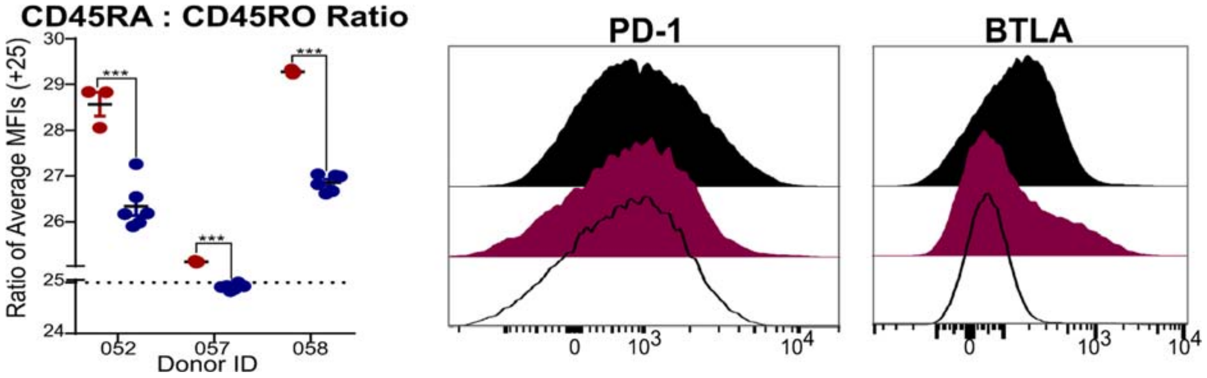

e

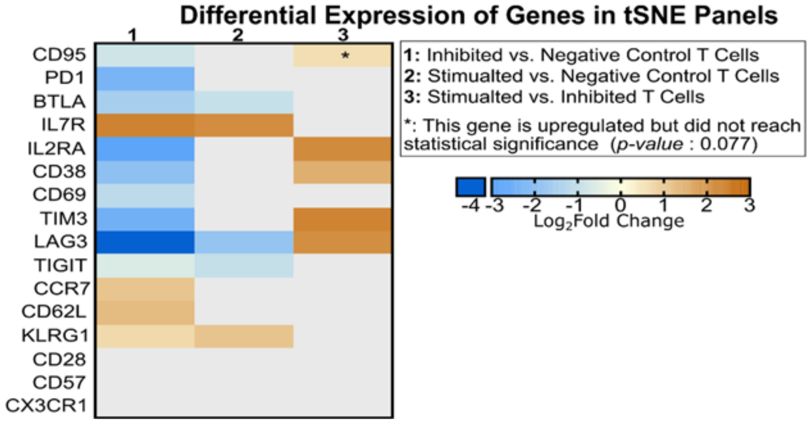

Figure S8 Continued | Other Markers Included in t-SNE Analyses and Gene Expression Data Complementing t-SNE Analyses t-distributed Stochastic Neighbor Embedding (tSNE) analysis of multi-color flow cytometry panels of naïve T cells activated with Dynabeads and incubated with (WT) IgG1-ICs or negative controls (Fc5 IgG1-ICs, monomeric IgG1, PBS only, etc.). Naïve T cells were sampled equally across all controls and donors (N=3 donors; 2 naïve CD8+ and 1 naïve CD4+). Red and blue arrows point to clusters of T cells treated with IgG1-ICs and Fc5 IgG1-ICs, respectively. Generations 6+ (Gen6+) represents T cell populations that have divided at least 5 times, whereas Generation 1 (Gen1) represents un-divided T cells. In addition to markers shown in main Figure 3, (**a**) Panel A examines markers associated with activation, exhaustion, and/or anergy. Gen1 T cells originating from IgG1-IC-treated or control-treated wells are shown in dark and light pink, respectively. Gen6 T cells originating from IgG1-IC-treated or control-treated wells are shown in dark and light grey, respectively. Heat-map t-SNE plots are shown for (i) CellTrace (ii) simultaneous stain for CCR7, CD62L, and CD28 (i.e. CCR7/CD62L/CD28 lump gate) (iii) CD57 (iv) simultaneous stain for CX3CR1 and KLRG1 (i.e. CX3CR1/KLRG1 lump gate) and (v) BTLA; (**b**) Panel B examines markers associated with differentiation status. Gen1 T cells originating from IgG1-IC-treated or control-treated wells are shown in dark and light purple, respectively. Gen6 T cells originating from IgG1-IC-treated or control-treated wells are shown in dark and light grey, respectively. Heat-map t-SNE plots are shown for select markers: (i) CellTrace (ii) CD127 (IL-2Rβ) (iii) CD45RO and (iv) CD28. (**c**) Heat-map t-SNE plots of non-stimulated naïve T cell are shown for all markers in Panels A and B. (**e**) Naïve CD8+ T cells activated with Dynabeads and incubated with either IgG1-ICs (red) or Fc5 IgG1-ICs (blue) were studied. However, IgG1- IC-treated T cells were first sorted to enrich for T cell populations stimulated by IgG1-ICs (roughly corresponding to Gen6+) and T cell populations inhibited by IgG1-ICs (Gen1). Fc5 IgG1-IC-treated T cells (control) are not sorted. Differential expression of genes of markers used in t-SNE analyses are shown to aid interpreting results associated with "lump gates" (e.g. TIGIT/TIM-3/LAG-3 on the APC channel). Genes are considered differentially expressed if the adjusted p-value < 0.05 and |fold change| is ≥ 1.5. (**e**) Univariate histograms for select markers from Panels A and B are shown. The black and fuchsia histograms represent Gen6+ and Gen1 T cells in all samples analyzed by t-SNE. Unfilled histograms pertain to non-stimulated T cells. The scatter dot plot shows the ratio of CD45RA:CD45RO expression (based on average MFI) for Gen6+ T cells that were incubated with IgG1-ICs (red) or controls (blue). Values below the dashed lines are negative (a common, unavoidable consequence of compensation). In this case, CD45RA expression for Donor 057 was much lower (negative CD45RA MFI) than CD45RO. Error bars represent the standard error of the mean (SEM) for each group. p-values were calculated using unpaired two-tailed t-tests using the Sidak-Holm correction for multiple comparisons. Asterisks *, **, and *** indicate p-values < 0.05, p-values < 0.01, and p-values < 0.0001, respectively.

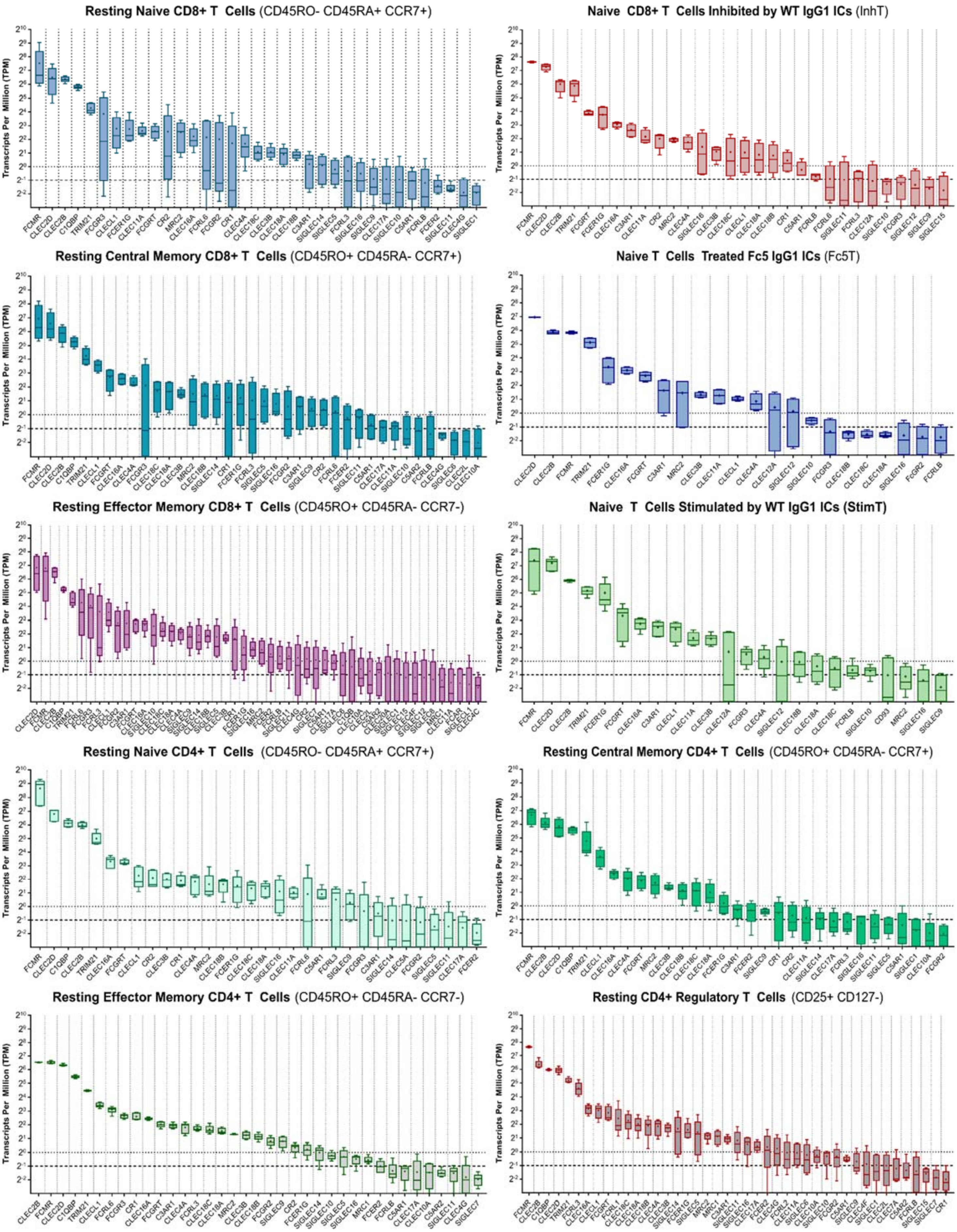

Figure S9 | Gene Expression of Select Receptors Including Lectins and Canonical/Non-Canonical FcRs

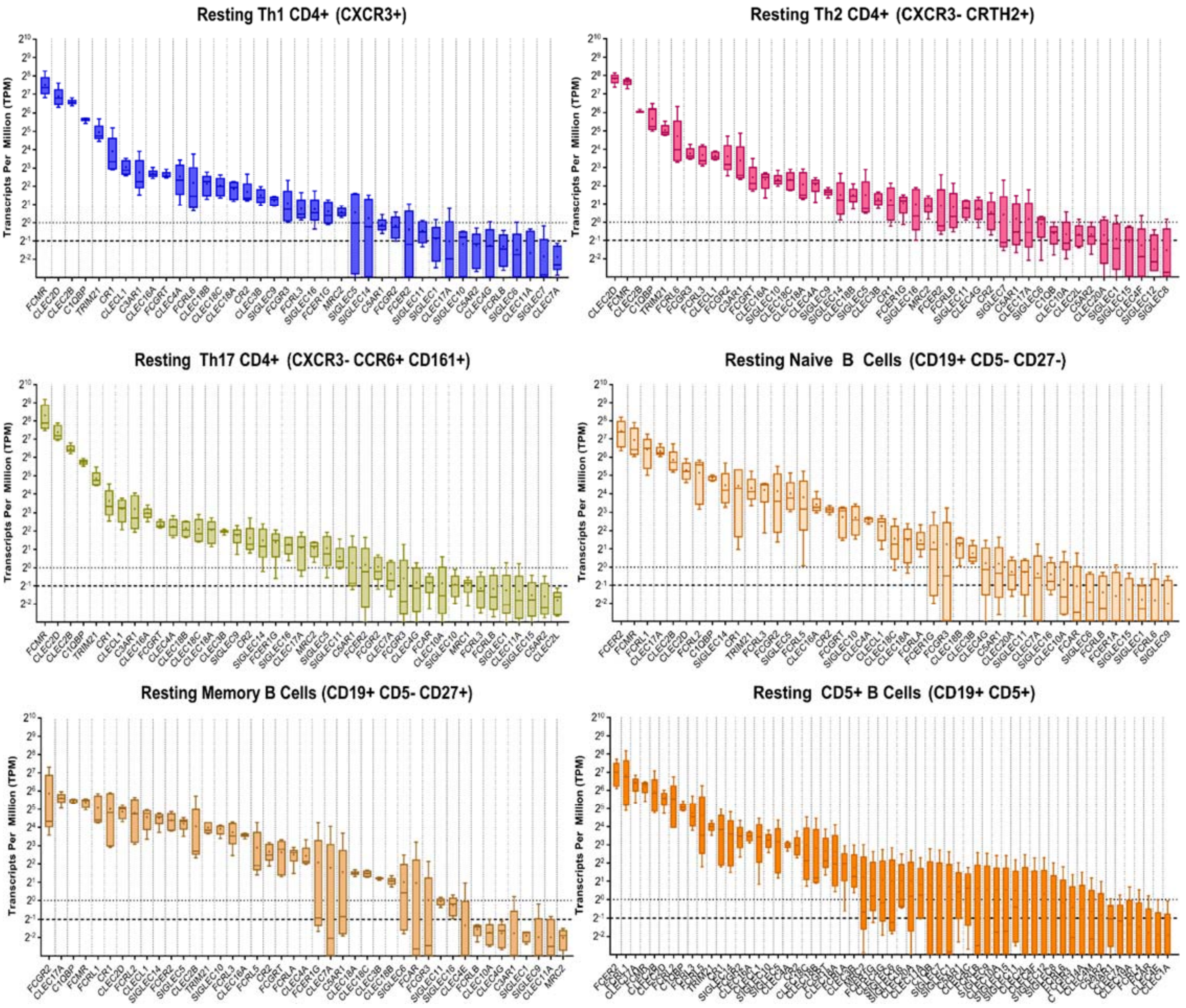

Figure S9 Conitued | Gene Expression of Select Receptors Including Lectins and Canonical/Non-Canonical Fc Receptors Transcripts per million (TPM) for various genes (including various C-type lectins, Sialic-acid-binding immunoglobulin-type lectins, Fc receptors, and Complement receptors) for various resting and activated lymphocyte subsets. RNA-Seq raw data for all "Resting" subsets is obtained from Ranzani et al[149]. The median (line), mean (dot), minimum/maximum(whiskers), and interquartile range (box) are shown. The dotted and dashed lines represent the TPM=1 (expressed) and TPM=0.5 (very low expression) thresholds.

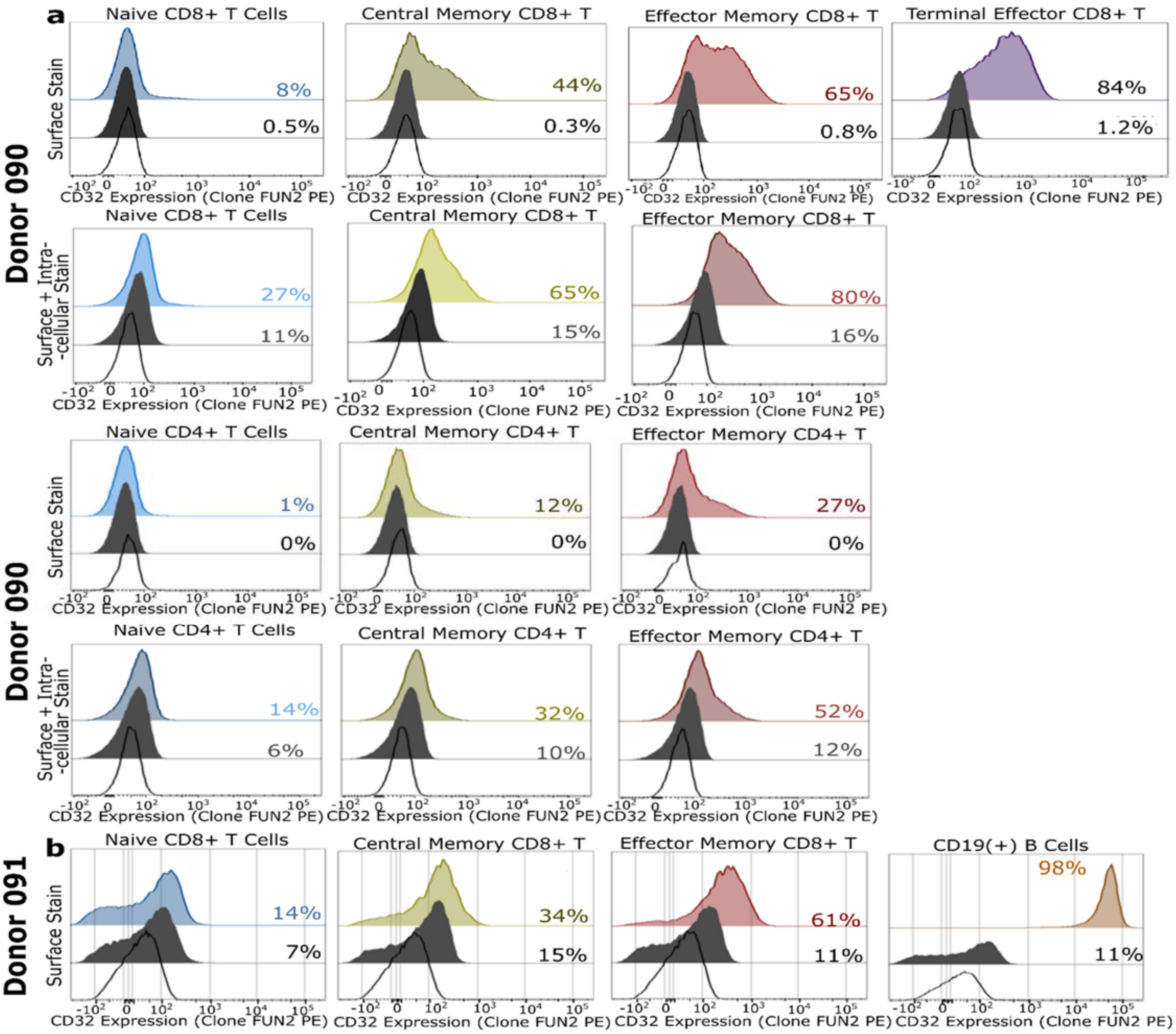

Figure S10 | FcγR Protein Expression is Observed Intracellularly and on the Cell Surface of Various T Cell Subsets (**a,b**) Various CD8+ and CD4+ T cell subsets were stained with anti-CD32 mAb (colored histograms) or isotype control (filled grey). Cells were either stained extracellularly (surface stain) or both intracellularly and extracellularly. FMOs are shown as unfilled histograms. Displayed percentages correspond to % positive events set relative to unstained cells. Note that "surface stain" only panels from Donor 090 are also presented in **Figure 6** but are shown here again alongside surface+intracellular stains for comparison.

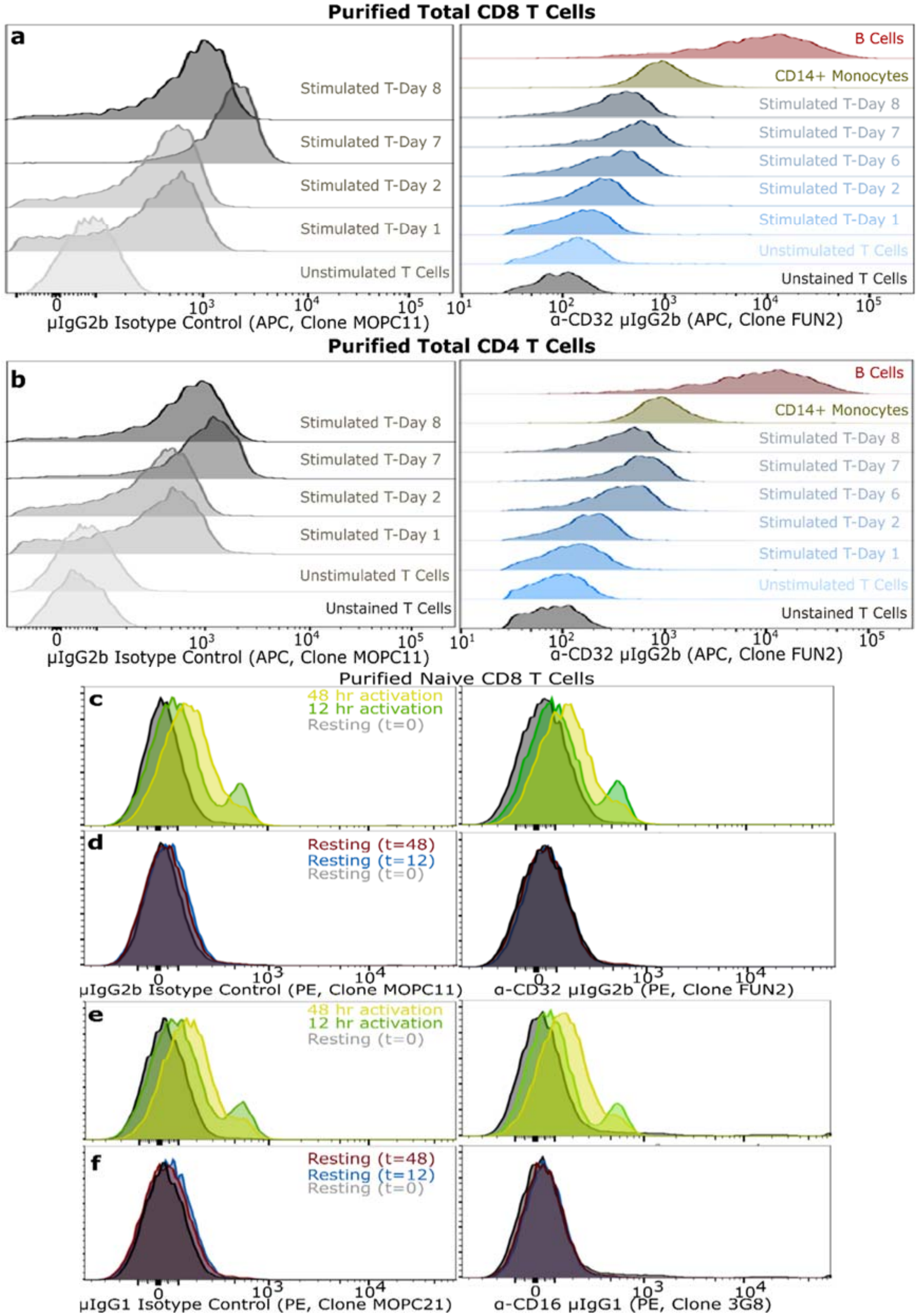

Figure S11 | FcR Surface Expression in T cells is Upregulated upon *In Vitro* Activation but Increasing Isotype Control Signal Obscures FcR Identity by Flow Cytometry Flow cytometry surface expression of FcγRII (CD32) on (**a**) Total CD8+ T cells (**b**) Total CD4+ T cells or (**c-f**) Naïve CD8+ T cells activated with anti-CD3/anti-CD28 Dynabeads *in vitro* and stained with anti-CD32 antibody, anti-CD16 antibody, or isotype control. Various time points after activation are shown. (**a-b**) Total CD4+ and CD8+ T cells are shown in grey or blue. As positive controls, resting B cells and monocytes are shown in red and yellow, respectively. (**c-f**) Naïve CD8+ T cells are shown in grey, red, blue, yellow or green based on activation status and/or various timepoints.

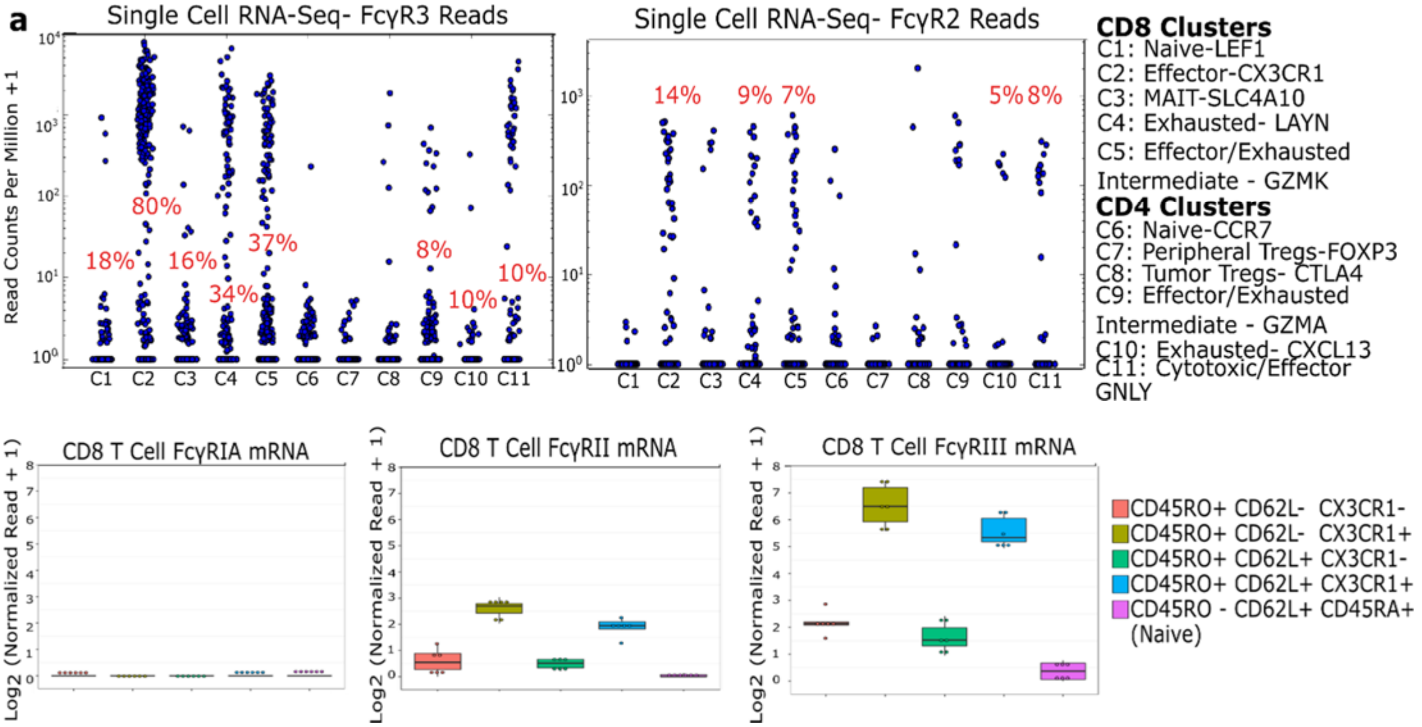

Figure S12 | FcγR Gene Expression is Observed in Other Datasets Involving T cells from Healthy and Patient Donors (**a**) Single-cell-RNA-Seq reads for FcγR3 (left panel) and FcγR2 (right panel) are shown for T cell clusters deduced from transcriptome data of hepatocarcinoma patients[150]. The percentage of cells with non-zero reads is shown in red for select clusters (**b**) Bulk RNA-Seq reads for various CD8+ T cell subsets. Raw data obtained from Bottcher *et al*[19].

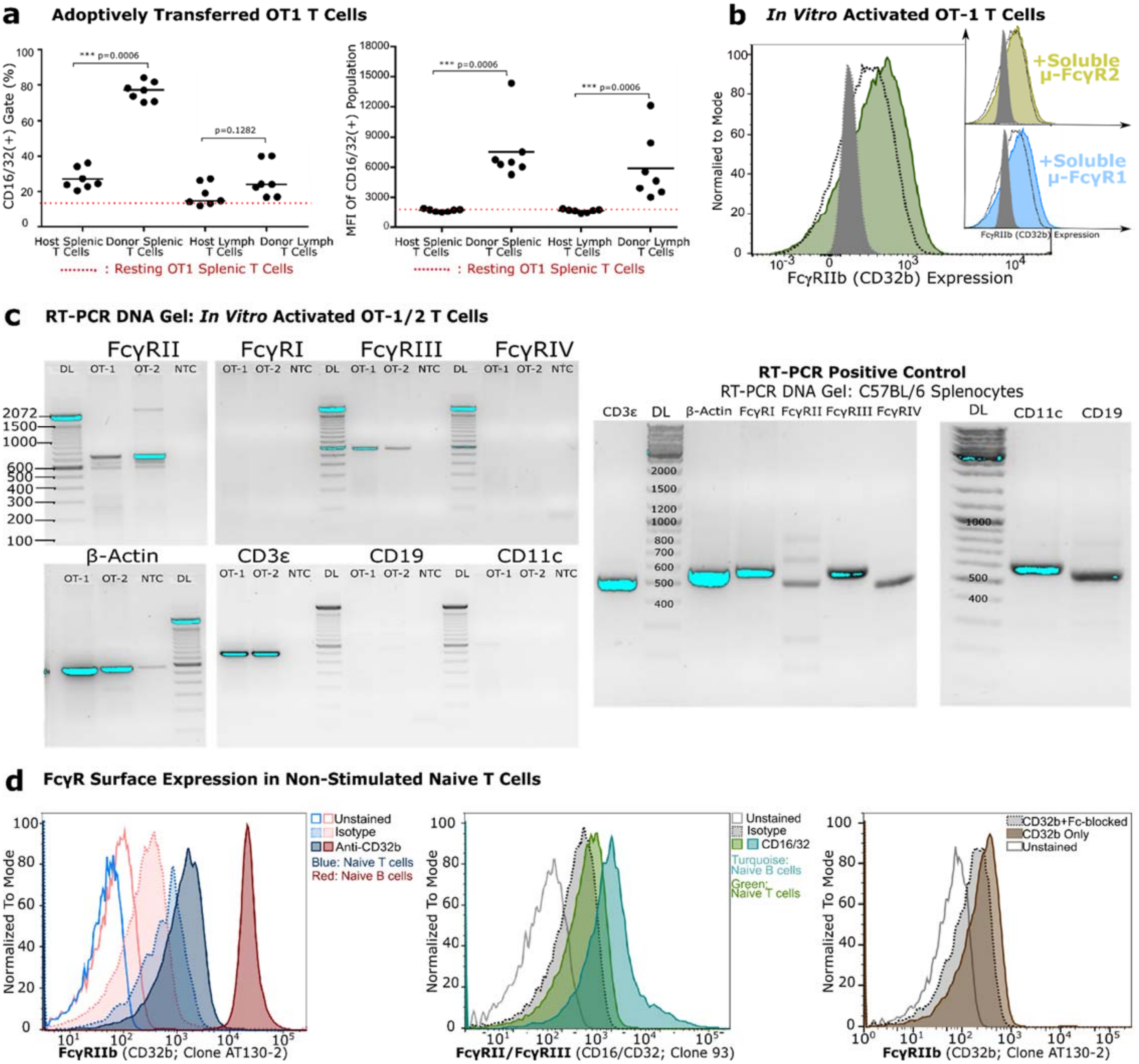

Figure S13 | FcγRs Are Also Expressed in Murine T Cells and Upregulated Upon *In Vivo* Activation (a) Flow cytometry results of CD45.2+ OT1 CD8+ T cells (donor) adoptively transferred into B16F10*OVA*-tumor-bearing CD45.1+ mice (host). The left panel shows the percent of CD16/32+ cells for endogenous host T cells or donor OT1 T cells recovered 9 days after adoptive transfer. The right panel shows the mean fluorescence intensity (MFI). The red line indicates values for resting, non-transferred splenic T cells from healthy OT1 mice. The % CD16/32(+) value is determined based on the FMO gate. p-values are calculated using the Mann Whitney t-test. (**b**) FcγRIIb expression of OT1 T cells activated *in vitro* with anti-CD3/CD28 Dynabeads and then stained in the presence of PBS (green), soluble non-fluorescent FcγRIIb (yellow, inlet panel), or irrelevant soluble non-fluorescent FcγRI (blue, inlet panel). Isotype controls are open, dashed histograms. Filled grey histograms are FMOs. (**c**) RT-PCR for sorted OT1 CD8+ and OT2 CD4+ T cells activated *in vitro* with anti-CD3/CD28 beads. "DL" stands for DNA ladder. The gene amplified is indicated over each gel. "NTC" stands for no-template-control. The DNA gel picture was uniformly color-inverted for easier visualization; turquoise bands indicate saturation. Positive controls are shown in the right DNA gel. (**d**) Flow cytometry expression of FcγRs in splenic Naïve T cells from wild-type C57BL/6 mice. Naïve B cells were used as positive controls. In the rightmost panel, before staining with anti-CD32b, naïve T cells were either pre-incubated with FcR-blocking-peptide solution (brown) or P

Supplementary Tables (S1-S10)

Type	Treatment	Components		Expected Binding[1] to Canonical FcRs
		Antigen	Antibody	
Immune Complexes	IgG1-ICs	TNP-BSA	α-TNP WT IgG1	FcγR1, FcγR2, and FcγR3
	Fc5 IgG1-ICs		α-TNP Fc5 IgG1	FcγR1 Only
	IgA1-ICs		α-TNP WT IgA1	FcαR1 Only
	IgE-ICs		α-TNP WT IgE	FcεR1 Only
Monomeric mAbs	IgG1 Isotype	TNP-BSA	α-TYRP1 WT IgG1	FcγR1 Only
	Fc5 IgG1 Isotype		α-TYRP1 Fc5 IgG1	FcγR1 Only
	IgA Isotype		α-TYRP1 WT IgA1	None
	IgE Isotype		α-TYRP1 WT IgE	FcεRI Only
	α-TNP IgG1	WT-BSA	α-TNP WT IgG1	FcγR1 Only
	α-TNP Fc5 IgG1		α-TNP Fc5 IgG1	FcγR1 Only
Antigen Only	TNP-BSA or WT BSA	WT BSA or TNP-BSA	NA	NA
PBS	α-CD3/CD28 Dynabeads Only	NA	NA	NA
	Non-Stimulated			

Table S1 | Immune Complexes and Other Experimental Controls Utilized in Functional Experiments Immune complexes were prepared by pre-incubating TNP-BSA and anti-TNP antibodies. Monomeric controls were generated by pre-incubating TNP-BSA and Isotype control antibodies or WT BSA and Anti-TNP antibodies. Antigen only controls pertain to TNP-BSA or WT-BSA and PBS (no antibodies). Expected binding to FcRs is based on ELISA results (**Figure S1**) in the relevant range of treatment concentration (1-50 μg/mL). Details pertaining to amounts and incubation time are provided (**Methods**).

Donor ID	T Cell Subset	% Undivided (Φ_1)		p-value	Percent Difference (%)
		Average Negative Controls	WT IgG1 ICs		
Donor 050	Naïve CD8+ T Cells	17.0 ± 8.4	79.7 ± 3.4	< 0.005	Mean: 200 ± 74 Range: 110-370
Donor 051		24.8 ± 3.2	70.5 ± 2.2		
Donor 062		15.6 ± 7.0	44.1		
Donor 052		26.1 ± 5.5	71.6 ± 1.5		
Donor 058		9.4 ± 0.8	20.0 ± 3.3		
Donor 017		21.9 ± 3.7	69.2 ± 5.8		
Donor 054		24.9 ± 2.0	65.0 ± 4.0		
Donor 050 *(1 year later)*		22.5 ± 2.7	65.9 ± 6.9		
Donor 017	Naïve CD4+ T Cells	31.2 ± 5.9	75.2 ± 2.3	< 0.005	Mean: 110 ± 54 Range: 50-190
Donor 060		13.4 ± 6.1	38.8 ± 1.1		
Donor 070		32.4 ± 6.9	66.8 ± 5.7		
Donor 057		17.0 ± 4.4	26.1 ± 3.4		
Donor 072		20.4 ± 2.1	35.8 ± 2.7	< 0.01	
Donor 051	Memory CD8+ T Cells	64.6 ± 4.3	73.4	0.053	Mean: 11 ± 3 Range: 7-14
Donor 070		56.5 ± 3.1	60.7 ± 0.8	0.07	
Donor 072		47.6 ± 3.2	53.2 ± 0.4	0.053	
Donor 017	Memory CD4+ T Cells	55.5 ± 0.8	52.8 ± 0.5	0.16	Mean: 8 ± 19 Range: 0-36
Donor 059		84.8 ± 3.4	85.4 ± 0.7	> 0.93	
Donor 070		49.4 ± 3.1	49.4 ± 4.2	> 0.99	
Donor 052		40.2 ± 2.4	54.8 ± 2.8	< 0.001	
Donor 017	Total CD8+ T Cells	49.3 ± 5.5	86.5	< 0.006	Mean: 61 ± 16 Range: 44-76
Donor 048		23.8 ± 3.3	39.1 ± 1.7		
Donor 050		60.7 ± 11.0	87.3		
Donor 017	Total CD4+ T Cells	35.9 ± 3.5	64.8	< 0.005	Mean: 56 ± 24 Range: 33-81
Donor 048		49.9 ± 4.9	66.3 ± 2.3		
Donor 050		43.7 ± 5.0	68.2		

Table S2 | T Cell Proliferation Experiments Mean values and standard deviations are tabulated for all proliferation experiments across T cell subsets and human donors. p-values are calculated using unpaired, two-tailed t-tests adjusting for multiple comparisons using the Sidak-Holm correction. Consistent standard deviation is not assumed unless an experiment pertains to a single-well assay (values with no standard deviations). The mean ± standard deviation and range of percent differences between IgG1-IC-treated T cells and controls are also shown.

Fluorophore/ Channel	Markers Associated with Activation, Exhaustion, and Anergy	Differentiation-Associated Markers
1. BV605	B3GAT1 (CD57)	IL-7R (CD127)
2. APCFire750	KLRG1 and CX3CR1	CD45RA
3. BV785		CD45RO
4. APC	LAG-3 and TIM-3 and TIGIT	Fas (CD95)
5. FITC	CCR7 and CD62L and CD28	CCR7 and CD62L
6. PE	IL-2Rα (CD25) and CD38 and CD69	CD28
7. BV711	PD-1	CD44
8. PE-Cy7	BTLA	
9. BV421	CellTrace Violet	CellTrace Violet

Table S3 | Multi-Color t-SNE Flow Cytometry Panels Surface markers used for t-SNE panels examining activation, exhaustion, and anergy (Panel A) differentiation (Panel B)[41,46,52–55]. Markers typically used to describe similar phenomena were lumped into one fluorophore channel (e.g. CCR7 and CD62L; IL-2Rα, CD69, and CD38; TIGIT, TIM-3, and LAG-3). Protocol details are included in the Methods section.

<table>
<tr><th rowspan="2">Genes</th><th colspan="3">InhT vs. StimT</th><th colspan="3">InhT vs Fc5T</th><th colspan="3">StimT vs Fc5T</th></tr>
<tr><th>Nominal p-value</th><th>Adjusted p-value</th><th>Log$_2$(FC)</th><th>Nominal p-value</th><th>Adjusted p-value</th><th>Log$_2$(FC)</th><th>Nominal p-value</th><th>Adjusted p-value</th><th>Log$_2$(FC)</th></tr>
<tr><td>BTLA</td><td>0.086</td><td>0.184</td><td>-0.55</td><td rowspan="9"><0.01</td><td rowspan="8"><0.01</td><td>-1.55</td><td rowspan="2"><0.01</td><td rowspan="2"><0.05</td><td>-1.01</td></tr>
<tr><td>CR1</td><td rowspan="5"><0.01</td><td rowspan="5"><0.05</td><td>2.50</td><td>6.83</td><td>4.34</td></tr>
<tr><td>CR2</td><td>4.68</td><td>4.26</td><td></td><td></td><td></td></tr>
<tr><td>CXCR3</td><td>-2.75</td><td>-2.04</td><td><0.01</td><td>0.017</td><td>0.71</td></tr>
<tr><td>CD49D</td><td>-1.74</td><td>-1.02</td><td><0.05</td><td>0.130</td><td>0.72</td></tr>
<tr><td>TBET</td><td>-1.76</td><td>-2.07</td><td></td><td></td><td></td></tr>
<tr><td>CD95</td><td rowspan="2"><0.05</td><td>0.077</td><td>-0.61</td><td>-0.87</td><td></td><td></td><td></td></tr>
<tr><td>FCGR2A</td><td>0.103</td><td>-3.76</td><td>-5.20</td><td></td><td></td><td></td></tr>
<tr><td>CD103</td><td></td><td></td><td></td><td>0.011</td><td>-0.66</td><td></td><td></td><td></td></tr>
<tr><td>IL2RB</td><td rowspan="16"><0.01</td><td rowspan="16"><0.05</td><td>-0.81</td><td>0.012</td><td>0.031</td><td>-0.63</td><td></td><td></td><td></td></tr>
<tr><td>FCGR3A</td><td>-2.34</td><td></td><td></td><td></td><td>0.060</td><td>0.201</td><td>1.47</td></tr>
<tr><td>GZMA</td><td>-3.24</td><td></td><td></td><td></td><td>0.065</td><td>0.212</td><td>1.85</td></tr>
<tr><td>GZMB</td><td>-3.19</td><td rowspan="7"><0.01</td><td rowspan="7"><0.01</td><td>-3.56</td><td></td><td></td><td></td></tr>
<tr><td>IFNG</td><td>-2.25</td><td>-2.65</td><td></td><td></td><td></td></tr>
<tr><td>CD11A</td><td>-0.94</td><td>-1.27</td><td></td><td></td><td></td></tr>
<tr><td>CD11B*</td><td>-4.14</td><td>-1.96</td><td><0.01</td><td><0.01</td><td>2.18</td></tr>
<tr><td>CD11C*</td><td>-4.32</td><td>-2.97</td><td>0.039</td><td>0.153</td><td>1.35</td></tr>
<tr><td>LAG3</td><td>-2.12</td><td>-4.00</td><td rowspan="2"><0.01</td><td rowspan="2"><0.05</td><td>-1.88</td></tr>
<tr><td>LAYN</td><td>1.47</td><td>-1.38</td><td>-2.85</td></tr>
<tr><td>PTK7</td><td>1.66</td><td>0.095</td><td>0.172</td><td>0.91</td><td></td><td></td><td></td></tr>
<tr><td>PKCθ</td><td>-0.60</td><td rowspan="9"><0.01</td><td rowspan="9"><0.01</td><td>-0.38</td><td></td><td></td><td></td></tr>
<tr><td>NFKB1</td><td>-0.62</td><td>-0.75</td><td></td><td></td><td></td></tr>
<tr><td>BCL2L1</td><td>-1.16</td><td>-1.36</td><td></td><td></td><td></td></tr>
<tr><td>FOXO1</td><td>1.16</td><td>0.75</td><td></td><td></td><td></td></tr>
<tr><td>EOMES</td><td>1.90</td><td>1.93</td><td></td><td></td><td></td></tr>
<tr><td>TSC1</td><td></td><td></td><td></td><td>1.03</td><td>0.071</td><td>0.225</td><td>0.63</td></tr>
<tr><td>IL12A</td><td></td><td></td><td></td><td>2.65</td><td><0.01</td><td><0.01</td><td>3.40</td></tr>
<tr><td>TCF7</td><td></td><td></td><td></td><td>1.09</td><td></td><td></td><td></td></tr>
<tr><td>LEF1</td><td></td><td></td><td></td><td>0.067</td><td>0.130</td><td>0.19</td><td></td><td></td><td></td></tr>
</table>

Table S4 | Differentially Expressed or Up/Downregulated Genes Mentioned In-Text DESeq2 was used to analyze differentially expressed genes (DEGs); genes were considered differentially expressed if the adjusted p-value < 0.05 and the |fold change (FC)| is ≥ 1.5 (i.e. the |Log$_2$(FC)| is ≥ 0.58496). Non-DEGs were considered upregulated/downregulated if either the adjusted or nominal p-value < 0.10. Grey blocks represent genes whose differences were not statistically significant (i.e. both nominal and adjusted p-value > 0.10)

Genes	InhT	Fc5T	StimT
BTLA	13.3	32.3	15.7
CR1	1.3	0.0	0.2
CR2	4.0	0.2	0.1
CXCR3	25.6	85.9	134.2
CD49D	58.7	99.8	155.4
TBET	2.8	9.7	7.9
CD95	24.8	36.0	28.9
FCGR2	0.1	0.3	0.2
CD103	6.7	10.2	7.0
IL2RB	84.2	133.6	85.8
FCGR3	0.4	0.4	1.4
GZMA	30.6	61.8	228.6
GZMB	15.5	141.3	112.5
IFNG	1.6	7.3	5.5
CD11A	183.5	375.3	284.2
CD11B*	0.7	2.1	9.0
CD11C*	1.8	11.7	26.4
LAG3	3.0	36.9	9.9
LAYN	20.6	44.5	6.1
PTK7	2.1	0.9	0.5
PKCθ	60.5	64.5	71.0
NFKB1	73.9	102.6	91.0
BCL2L1	16.4	34.0	29.0
FOXO1	59.5	29.7	21.2
EOMES	2.8	0.6	0.6
TSC1	14.7	6.1	8.7
IL12A	1.1	0.1	1.2
TCF7	210.4	83.1	99.1
LEF1	263.2	194.0	185.8

Table S5 | Average Transcripts Per Million (TPMs) for Genes Mentioned In-Text

Note that TPMs are not well-suited for comparisons across samples (DESeq2 analysis is used for differential expression across samples) only within genes of a sample. TPM<0.5 indicates weak or no expression. TPM > 1 indicates expression.

Comparison	Hallmarks	Normalized Enrichment Score (NES)	Nominal p-value	FDR-Adjusted q-value
InhT vs. Rest	Wnt/β-Catenin Signaling	1.74	0.002	0.004
	Hedgehog Signaling	1.52	0.024	0.028
	Interferon-α Response	1.51	0.009	0.020
	TGF-β Signaling	1.42	0.046	0.041
StimT vs. Rest	Oxidative Phosphorylation	2.86	0.00	0.00
	Myc Targets V1	2.70	0.00	0.00
	Myc Targets V2	2.46	0.00	0.00
	Reactive Oxygen Species Pathway	2.18	0.00	0.00
	DNA Repair	1.85	0.00	0.001
	Fatty Acid Metabolism	1.60	0.00	0.008
	Allograft Rejection	1.58	0.00	0.009
	Peroxisome	1.47	0.005	0.022
	mRTORC1 Signaling	1.38	0.007	0.040
	PI3K/Akt/mTOR Signaling	1.35	0.027	0.045
	Adipogenesis	1.22	0.058	0.135
Fc5T vs. Rest (Top 15)	E2F Targets	2.98	0.00	0.00
	G2M Checkpoints	2.82	0.00	0.00
	Cholesterol Homeostasis	2.63	0.00	0.00
	mTORC1 Signaling	2.58	0.00	0.00
	Mitotic Spindle	2.50	0.00	0.00
	Hypoxia	2.21	0.00	0.00
	TNF-α Signaling via NFKB	2.12	0.00	0.00
	Glycolysis	1.92	0.00	0.00
	Spermatogenesis	1.87	0.00	0.00
	Estrogen Late Response	1.86	0.00	0.00
	Adipogenesis	1.82	0.00	0.00
	IL2/STAT5 Signaling	1.79	0.00	0.00
	Androgen Response	1.78	0.00	0.00
	Apical Junction	1.76	0.00	0.00
	Apoptosis	1.74	0.00	0.00
	E-Cadherin Nascent Pathway	1.58	0.015	0.053

Table S6 | GSEA Analyses Reveal Other Hallmarks Enriched in Inhibited and Stimulated T cell Populations (**a-c**) In addition to pathways and hallmarks highlighted in main **Figure 5**, GSEA analyses reveal other potentially meaningful enrichments. Gene set enrichment analysis (GSEA) identifies pathways that correlate to the enrichment of genes across the InhT, StimT, and Fc5T groups. "Rest" represents any two of these groups. The FDR q-value and the normalized enrichment score (NES) are tabulated.

Gene	Gene Expression in ***Any*** T Cell Subset	Known Binding to Fc		
		IgG	IgA	IgE
**FcγRI**	+	+	-	-
**FcγRII**	+	+	-	-
**FcγRIII**	+	+	-	-
FcεRI	-	-	-	+
**FcεRII**	+	+/-	-	+
FcαRI	-	-	+	-
FcRL4	-	-	+	-
FcRL5	-	+	-	-
**TRIM21**	+	+	+	+
MBL2	-	+	+	-
**MRC2**	+	+/-	+/-	?
DC-SIGN	-	+/-	+	?
Dectin-2	-	+	?	?
**Dectin-1**	+	+	+	?
**CD22**	+	-	?	+
pIgR	?1	-	+	-
**FcμR (IgM)**	+	-	-	-
**FcαμR**	?	-	+	-

Table S7 | Only FcγRs exclusively bind IgG and are expressed in any T cell subset This table lists genes pertaining to receptors with reported binding (or non-binding) to IgG, IgA, and/or IgE (refs). Gene expression is based on RNA-Seq data presented in this work or any other reported literature (refs). Receptors with observed gene expression in any T cell subset and known binding to either IgG, IgA, or IgE are highlited in red.

Sample ID	Sample Type	Sample Date	Number of Cells (Millions)
1015014 PBMC Lab08-0295 DAL	Not Specified	05/17	10
955943 PBMC Lab08-295 DAL	Not Specified	05/17	4
PXX CXX DOR, M Osteosarcoma	Osteosarcoma	05/14	4.85
PBMC OS0001NK Osteosarcoma PT CD	Osteosarcoma	09/09	3
PBMC 2510 GCR CD	Gastrointestinal Cancer	03/10	20
PXX CXX AL, S, G BL-Osteo CD	Osteosarcoma	05/10	10
PXX CXX VOR, A Osteosarcoma PT KT	Osteosarcoma	05/14	4.5
PXX CXX SAN, J Osteosarcoma PT KT	Osteosarcoma	05/15	4.79
PBMC 201195 GCR CD	Gastrointestinal Cancer	03/10	5
PBMC 209568 GCR CD	Gastrointestinal Cancer	03/10	10
ALL-9 DAL	Acute Lymphoblastic Leukemia	06/07	1000
ALL-11 BMNC	Acute Lymphoblastic Leukemia	02/26	5.00
AML7 DAL	Acute Myeloid Leukemia	12/23	50
PXXX CXXX ALK, A KT	Not Specified	05/09	*Not Counted*
PBMC GCR 8269933	Gastrointestinal Cancer	02/27	10

Table S8 | Human Cancer Patient Information This table lists the known information associated with cancer patient PBMCs provi050ded by D. Lee of M.D. Anderson

Gene	Forward Primer (5'-3')	Reverse Primer (5'-3')	Expected Band Size
β-Actin	GAACATGGCATT GTTACCAAC	GCATCGGAACCGCTC GTTGCC	558 bp
CD3ε	GACGATGCCGAGAACA TTGAATACAAAGTC	GACTGCTCTCTGAT TCAGGCCAGAATA	504 bp
CD11c	CTACTGAGTTCATCATT CAAGCAGAGCCA	CTCAATATCCTTCAAAGCATC AAAGTTCTCCAC	600 bp
CD19	CTCACTGTGGC ACCCGGCTC	CAGCAGCCCAACG CTGAGCATG	550 bp
FcγRI	GCATCCCAGAGG CCAGTTTTCAGG	CCTCCGTGGCTACC TCACACC	582 bp
FcγRII	CGGAGCCAGGT CCAAGCCAG	CAGGGCTTCGGG ATGCTTGAGAAG	723 bp Isoform B1<br>639 bp Isoform B1',<br>582 bp Isoform B2
FcγRIII	GGGACCCACAAC CCTGGGAAC	ATTGACAGGGACT TCCTCCAGTAATCC	584 bp
FcγRIV	TGCCAACTATGTCAT CCAAAGTGCCAGA	TCATAGTCAGCCAC AGGACTTTGAAGAC	498 bp
FcγRII *all isoforms*	CGGAGCCAGGT CCAAGCCAG	CTTCCTAGACTTCCTT TGCAGTAGTAGTC	323 bp

Table S9 | Murine T Cell RT-PCR Primer Design

Antibody	Sequence (5’-3’)
**Anti-TNP**<br>Clone 7B4<br>*Heavy Chain*	GAACAGATCCAGTTGGTACAGTCTGGACCTGAGGTGAAGGAGCCTGGAGA<br>GACAGTCAGGATCTCCTGCAAGGCTTCTGGATATACCTTCACAGCCCATGG<br>AATGGGCTGGGTGAAACAGGCTCCAGGAAAGGGTTTAAGGTGGATGGGCT<br>GGATAAACACCTACTCTGGAGTGCCAGCATATGTTGATGACTTCAAGGGAC<br>GGTTTGCCTTTTATCTGGAAACCTCTGCCAGCACTGTCTATTTGCAGATCAA<br>TAACGTCAAAGATGAAGACACGGCTACATATTTCTGTGGAAGATGGGCTAT<br>GGTTACGACGTGCTTTGACACCTGGGGCCAAGGCACCACTCTCACCGTGAG<br>CTCA
**Anti-TNP**<br>Clone 7B4<br>*Light Chain*	TCAATGTCCAGAGGAGAAAATGTGGTCACCCAGTCTCCAGGAATCATGTCT<br>GCATCTCCAGGGGACAAGGTCACCATGACCTGCAGGGCCAGCCCAAGTGTA<br>ACTTCCAGTCACTTGCACTGGTATCAGCAGAAGTCAGGTGGCTCCCCCAAA<br>CTCTGGATTTATAGCACATACAACTTGGCTTCTGGGGTCCCTGGTCGCTTCA<br>GTGGCAGTGGGTCTGGGGCCTCTTACTCTCTCACAATCAGCAGTGTGGAGG<br>CTGAAGATGCTGCCACTTATTACTGCCAGCAGTACAGAAGTTATTCAGTCA<br>CGTTCGGAGGGGGGACCAAGCTGGAGATCAAACGA
**Anti-TYRP1**<br>Clone 20D7S<br>*Heavy Chain*	CAGGTGCAGCTGGTCCAATCTGGGTCTGAGTTGAAGAAGCCTGGGGCCTCA<br>GTGAAGATTTCCTGCAAGGCTTCTGGATACACCTTCACTAGCTATGCTATGA<br>ATTGGGTGCGACAGGCCCCTGGACAAGGGCTTGAGTCTATGGGATGGATCA<br>ACACCAACACTGGGAACCCAACGTATGCCCAGGGCTTCACAGGACGGTTTG<br>TCTTCTCCATGGACACCTCTGTCAGCACGGCATATCTGCAGATCAGCAGCCT<br>AAAGGCTGAGGACACTGCCATATATTACTGTGCGCCCCGATATAGCAGCAG<br>CTGGTACCTTGATTACTGGGGCCAGGGAACCCTGGTCACCGTGTCCTCA
**Anti-TYRP1**<br>Clone 20D7S<br>*Light Chain*	GAGATCGTGCTGACACAGAGCCCTGCCACCCTGTCTCTGAGCCCTGGCGAA<br>AGAGCCACCCTGAGCTGTAGAGCCAGCCAGAGCGTGTCCAGCTACCTGGCC<br>TGGTATCAGCAGAAGCCCGGCCAGGCTCCCCGGCTGCTGATCTACGATGCC<br>AGCAATAGAGCCACCGGCATCCCCGCCAGATTTTCCGGCTCTGGCAGCGGC<br>ACCGACTTCACCCTGACCATCAGCTCCCTGGAACCCGAGGACTTCGCCGTG<br>TACTACTGCCAGCAGCGGAGCAACTGGCTGATGTACACCTTCGGCCAGGGC<br>ACCAAGCTGGAAATCAAGCGGACAGTGGCCGCTCCCA
**Anti-CD32b**<br>2B6 Chimera<br>*Heavy Chain*	CAGGTTCAGCTGGTGCAGTCTGGAGCTGAGGTGAAGAAGCCTGGGGCCTCA<br>GTGAAGGTCTCCTGCAAGGCTTCTGGTTACACCTTTACCAACTACTGGATAC<br>ACTGGGTGCGACAGGCGCCTGGACAAGGGCTTGAGTGGATGGGAGTGATT<br>GATCCTTCTGATACTTATCCAAATTACAATAAAAAGTTCAAGGGCAGAGTC<br>ACCATGACCACAGACACATCCACGAGCACAGCCTACATGGAGCTGAGGAG<br>CCTGAGATCTGACGACACGGCCGTGTATTACTGTGCGAGAAACGGTGATTC<br>CGATTATTACTCTGGTATGGACTACTGGGGGCAAGGGACCACGGTCACCGT<br>CTCCTCA
**Anti-CD32b**<br>2B6 Chimera<br>*Light Chain*	GATATTCAAATGACCCAAAGCCCGTCTTCTTTAAGCGCGTCTGTCGGTGATC<br>GCGTGACCATCACGTGTCGTGCGAGCCAAGATGTTAATACGGCGGTGGCCT<br>GGTATCAACAAAAACCGGGTAAAGCCCCGAAGCTGTTAATCTACAGCGCCA<br>GCTTTCTGTACTCTGGCGTCCCGAGCCGCTTTTCTGGCAGCCGCAGCGGTAC<br>GGACTTCACGCTGACCATTAGCAGCCTGCAGCCGGAGGATTTCGCCACCTA<br>TTATTGTCAGCAGCACTACACCACCCCGCCAACCTTTGGCCAGGGTACGAA<br>AGTGGAGATTAAACGC

Table S10 | Recombinant Antibody Sequences

Methods for Supplemental Murine Studies

OT-I/II T Cell *In Vitro* Activation

Spleens were excised from OT-I and OT-II mice. T cells were then magnetically isolated from processed splenocytes (STEMCELL 19851). T cells were cultured at 1M/mL in complete medium (10ng/mL rμ-IL2) and activated with anti- mouse CD3/CD28 dynabeads (Thermo). Four days later, activated T cells were washed and processed for further analysis.

Mouse RT-PCR Assay

To ensure high purity for RT-PCR, activated OT T cells were further sorted to remove any impurities (CD19, B220, CD11c, Ly6G) and only process TCRβ/CD4(+) OT-2 and TCRβ/CD8(+) OT-1 T cells. Approximately 1M sorted OT1 and OT2 T cells were processed for RNA extraction (Qiagen). 580 ng of OT1 total RNA and 310 ng of OT2 total RNA were processed to amplify cDNA using pre-designed primers and SuperScript® III One-Step RT-PCR System with Platinum® Taq DNA Polymerase (Thermo). The cDNA production step was set at 55°C for 35 minutes. That was followed by DNA denaturation (120s, 94°C) and 32 PCR cycles- denaturation (15s, 94°C), annealing (30s, 55°C), extension (35s, 68°C). Employed primer sequences and expected band sizes are provided (**Table S3**).

OT-1/ B16F10OVA In Vivo Mice Study

CD45.1(+) WT C57BL/6 mice were inoculated with 10^5 B16F10***OVA*** cells subcutaneously (s.c.). Two weeks later, CD45.2(+) OT1-OVA-specific CD8 T cells were adoptively transferred into tumor-bearing mice. Nine days after transfer, mice were euthanized and CD45.1(+) host T cells along with CD45.2(+) donor OT1 T cells were isolated from spleens and lymph nodes. Host and donor T cells were stained with anti-mouse CD16/CD32 (Clone 93).

FcγR Expression by Flow Cytometry

Anti-mouse CD32b (clone AT130-2) and its matching IgG2a isotype control (clone eBM2a) were purchased from eBioscience. Anti-mouse CD16/CD32 (clone 93) was purchased from Biolegend. Isotype control brightness and final concentrations were matched to that of FcγR-staining mAbs before staining. Where indicated, an FcR-blocking peptide solution was used (Innovex Biosciences). Activated OT T cells were stained for FcγRIIb with anti-CD32b mAb PE (Clone AT130-2) or isotype (clone eBM2a) and analyzed by FACS. To ensure that observed fluorescence shifts were indeed due to FcγRIIb binding, aliquots of anti-CD32b were pre-incubated with either soluble his-tagged- murine FcγRIIb, irrelevant soluble his-tagged-murine FcγRI, or DPBS. The soluble receptors (2-3 mg/mL) were added in molar excess (~50 μg HIS- FcγR in ~20uL DPBS) to the staining antibody (~2 μg in ~10uL) and incubated on ice for at least 30 minutes. Using those stocks, equal molar amounts of antibody were used to stain cells before FACS analysis. Cells were pre-blocked with rat serum for 20 minutes on ice. Antibody staining was done in 20%

rat serum DPBS. Recombinant HIS-tagged FcγRs were produced in Expi293F cells, as described earlier, and purified by Nickel-NTA column chromatography (Thermo).

Supplementary Attachments

Supplementary Attachment 1. Proliferation Analysis Template. See separate excel file.
